# Excitatory glycine receptors control ventral hippocampus synaptic plasticity and anxiety-related behaviors

**DOI:** 10.1073/pnas.2501118122

**Published:** 2025-09-09

**Authors:** Lara Pizzamiglio, Elise Morice, Cécile Cardoso, Simon Bossi, Caroline Mailhes-Hamon, Moritz von Heimendahl, Gabrielle Girardeau, Pierre Paoletti

**Affiliations:** ^a^Institut de Biologie de l’Ecole Normale Supérieure, Ecole Normale Supérieure, Université Paris Sciences et Lettres, Centre National de la Recherche Scientifique, Institut National de la Santé et de la Recherche Médicale, Paris 75005, France; ^b^Institut du Fer à Moulin, Institut National de la Santé et de la Recherche Médicale, Sorbonne Université, Paris 75005, France; ^c^Central Nervous System Diseases Research, Boehringer Ingelheim Pharma GmbH & Co. KG, Biberach an der Riss 88397, Germany

**Keywords:** neurotransmission, NMDA receptors, glycine, GluN3A, ventral hippocampus

## Abstract

Excitatory glycine receptors (eGlyRs), composed of the glycine-binding NMDA receptor subunits GluN1 and GluN3A, have recently emerged as a novel neuronal signaling modality that challenges the traditional view of glycine as an inhibitory neurotransmitter. Unlike conventional GluN1/GluN2 NMDARs, the distribution and role of eGlyRs remain poorly understood. Here, we show that eGlyRs are highly enriched in the ventral hippocampus (VH) and confer distinct properties on this brain region. eGlyRs display a massive expression in both VH CA1 pyramidal cells and SST- and PV-positive interneurons, whereas in the dorsal hippocampus (DH) pyramidal cells lack these receptors. eGlyRs mediate excitatory tonic currents and control VH network excitability. They are also responsible for the attenuated long-term potentiation (LTP) in the VH compared with the DH, providing a molecular basis for this difference. Furthermore, eGlyRs are required for regulation of VH LTP by corticosterone, pointing to eGlyRs as mediators of the neuroendocrine stress response. Consistent with this pervasive influence in the ventral division of the hippocampus, eGlyRs contribute to the modulation of anxiety-related behaviors. Our work identifies eGlyRs as key players in VH circuitry and function, demonstrating their intimate association with brain regions that control internal states and emotional processing.

## Significance

The hippocampus shows a deep anatomical and functional differentiation across the longitudinal axis, with the dorsal subregion involved in primarily cognitive function and the ventral subregion in emotions and anxiety. The molecular basis of this segregation remains poorly understood. In this study, we demonstrate that GluN1/GluN3A excitatory glycine receptors (eGlyRs)—unconventional NMDARs insensitive to glutamate and activated by glycine only—are strongly enriched in the ventral hippocampus (VH) where they influence network excitability, synaptic plasticity, and its regulation by the stress hormone corticosterone. We also show that VH eGlyRs contribute to anxiety behaviors. Overall, our findings reveal a critical influence of eGlyRs in the physiology and behavioral encoding of the VH.

Understanding how the activity of individual neurons is ultimately related to behavior in both normal and disease states is a central quest in neuroscience. For that purpose, it is essential to identify and dissect the different signaling mechanisms that drive the activity of single neurons. In the last years, a new type of neuronal NMDA receptor (NMDAR) composed of the GluN1 and GluN3A subunits (GluN1/GluN3A receptors) has been identified, revealing a previously unknown intercellular signaling mechanism in the brain. Although structurally and phylogenetically related to NMDARs and other tetrameric ionotropic glutamate receptors (iGluRs) (1–5), GluN1/GluN3A receptors depart from conventional GluN1/GluN2 NMDARs by their insensitivity to glutamate and reliance on the exclusive binding of glycine for their activation. GluN1/GluN3A receptors therefore form glycine-gated cationic channels, also named excitatory glycine receptors (eGlyRs), in opposition to glycine-gated anionic channels also known as pentameric glycine receptors (GlyRs). GlyRs along with GABA_A_ receptors provide the bulk of inhibitory neurotransmission in the CNS (6, 7). Besides their unresponsiveness to glutamate, eGlyRs differ from conventional NMDARs by their low Ca^2+^ permeability, greatly reduced Mg^2+^ block, as well as insensitivity to classical NMDAR antagonists such as D-AP5 and MK-801 (8–10). Equipped with four glycine sites (2 on GluN1 and 2 on GluN3A subunits), eGlyRs also display an atypical gating mechanism whereby glycine acts both as an agonist and a functional antagonist, as manifested by a bell-shaped dose–response curve (8–12). Glycine binding to GluN3A triggers channel opening, whereas glycine binding to GluN1 causes autoinhibition, thus resulting in rapid and pronounced receptor desensitization. First characterized over twenty years ago in heterologous expression systems (9), the existence of functional eGlyRs in the CNS has long remained elusive. This situation changed radically with the identification that the compound CGP-78608, a known GluN1 glycine-site antagonist and inhibitor of conventional NMDARs, massively and potently potentiates eGlyRs (11). Leveraging the power of CGP-78608, eGlyRs were shown to exist in the juvenile brain (11, 13, 14), but also in several discrete regions and neuronal populations of the adult brain. Functional eGlyRs were found in principal cells of the adult medial habenula (MHb) and basolateral amygdala (BLA) where they participate in the control of aversive behaviors (15) and stabilization of fear memories (16), respectively. Operational eGlyRs are also strongly enriched in somatostatin positive interneurons (SST-INs) of the adult somatosensory neocortex where they control the behavioral-state-dependent modulation of cortical circuits (16). In these cells, eGlyRs localize diffusely at synaptic and extrasynaptic sites, and generate tonic currents by sensing ambient extracellular glycine (16, 17). Despite these recent progresses, our understanding of eGlyRs remains limited and lags far behind that of conventional NMDARs. Expression studies indicate that, beyond the MHb, BLA, and neocortex, GluN3A is also strongly but unevenly expressed in the adult hippocampus (18–21), translating into preferential eGlyR expression in the ventral division of the hippocampus (22). Here, using a multiscale approach, we establish the cellular distribution, activation modality, and role of eGlyRs in adult hippocampal circuits and their implication in hippocampal-related behaviors. We present evidence that eGlyRs establish a signaling modality in the adult hippocampus with anatomical and functional features strikingly different from conventional NMDARs. We demonstrate that eGlyRs are strongly enriched in the ventral hippocampus (VH), thereby conferring specific properties to this region which makes it distinctive from the dorsal hippocampus (DH). We also show that eGlyRs in the VH are tightly linked with cellular and behavioral mechanisms encoding, and regulating, anxiety, and stress-related memories. Our work, together with previous studies (15, 16), points to eGlyR signaling as an important modality of intercellular communication in the adult brain, with strong implications in neuropsychiatric-relevant regions (BLA, MHb, VH) serving as key nodes for emotional processing.

## Results

### eGlyRs Segregate in CA1 Pyramidal Neurons of the Adult VH.

**Fig. 1.**
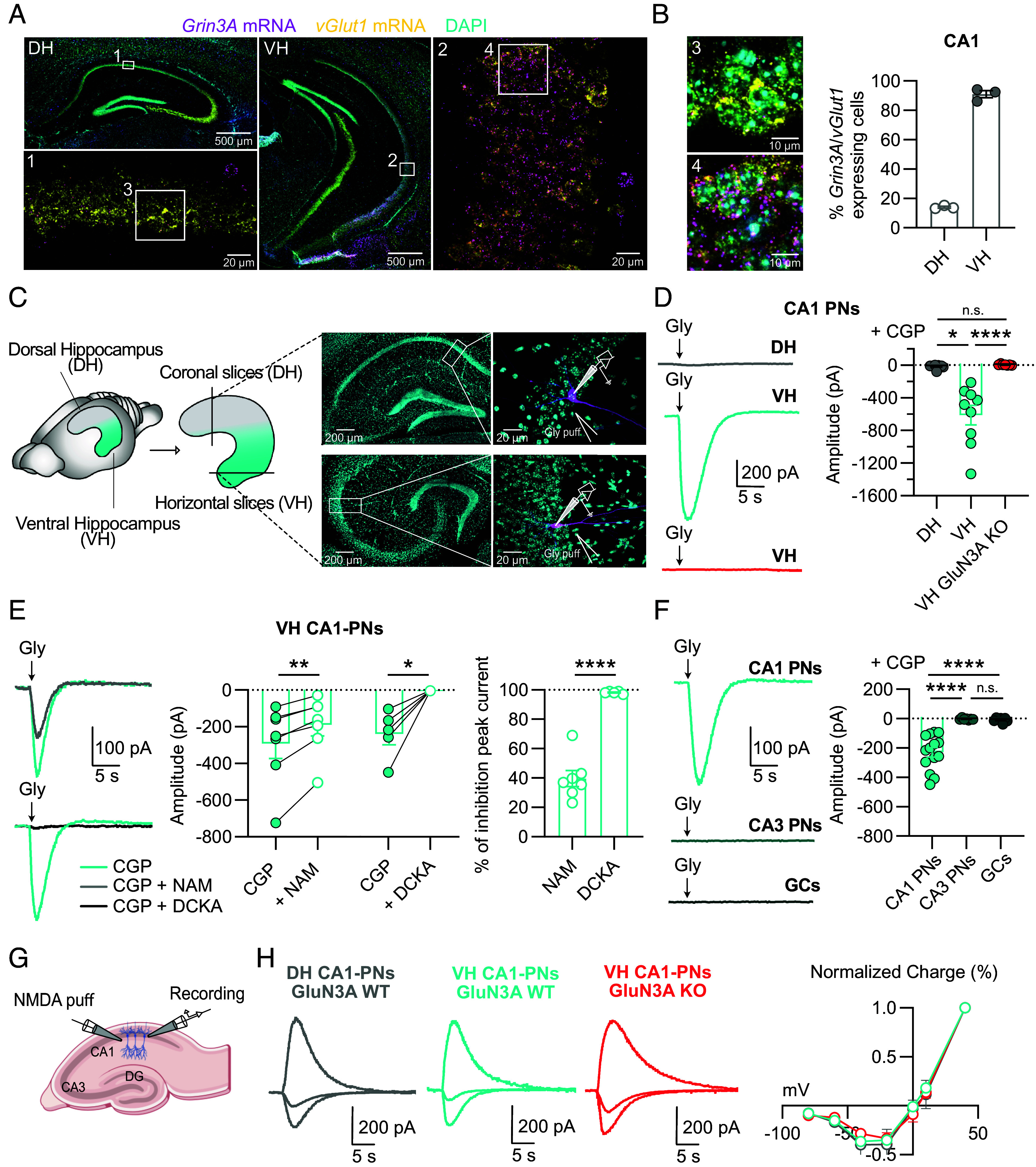
In adult CA1 pyramidal neurons (PNs), eGlyRs segregate in the ventral hippocampus (VH). (*A*) Representative coronal sections of the dorsal hippocampus (DH) and VH labeled by RNAscope for *Grin3a* mRNA (magenta), *vGlut1* mRNA (yellow), and DAPI (cyan). (1, 2) High-magnification images of the boxed areas in DH (1) CA1 and VH (2) CA1 hippocampal subregions. (*B*) *Left*: Higher-magnification images (3, 4) of the boxed areas in 1 and 2. *Right*: Quantification of the percentage of *Grin3a* mRNA-positive cells in CA1 of the DH and VH which also express *vGlut1* (N = 3 animals per condition). (*C*) Representative images of patched PNs and location of the glycine puff pipette in CA1 layer of the DH and VH. (*D*) Puffs of glycine (10 mM) in the presence of CGP (1 µM) induce inward currents (−65 mV) in CA1 PNs only in the VH but not in GluN3A-KO CA1 PNs (DH (n = 9) vs VH (n = 9), *P* = 0.0118; VH vs VH GluN3A KO (n = 7), *P* < 0.0001, Kruskal–Wallis followed by Dunn’s multiple comparisons test). (*E*) Pharmacological inhibition of glycine-evoked currents (glycine 1 mM, −65 mV) by NAM (30 µM) or DCKA (500 µM) in the presence of CGP (1 µM) [CGP vs CGP + NAM (n = 7), *P* = 0.0044; CGP vs CGP + DCKA (n = 5), *P* = 0.0161, Paired *t* test]. *Right*: Percentage of inhibition of the peak current [NAM (n = 7) vs DCKA (n = 5), *P* < 0.0001, Unpaired *t* test]. (*F*) Glycine puff (1 mM) experiments in the presence of CGP (1 µM) in CA1, CA3, and granule cells (GCs) of the dentate gyrus of the VH (CA1 PNs (n = 14) vs CA3 PNs (n = 7), *P* < 0.0001; CA1 PNs vs GCs (n = 7), *P* < 0.0001, Ordinary one-way ANOVA followed by Holm–Sidak’s multiple comparisons test). (*G*) NMDA puff experiments in the CA1 layer. (*H*) Representative traces (−60, −20, +40 mV) and current–voltage relationship (I–V) of currents elicited by pressure-applied NMDA (1 mM) in WT CA1 PNs of the DH and VH WT PNs and GluN3A-KO CA1 PNs of the VH [−60 mV: WT VH (n = 9) vs WT DH (n = 6), *P* = 0.9662; WT VH vs KO VH (n = 7), *P* = 0.7450; WT DH vs KO VH, *P* = 0.1419; −80 mV: WT VH (n = 9) vs WT DH (n = 6), *P* > 0.9999; WT VH vs KO VH (n = 5), *P* > 0.9999; WT DH vs KO VH, *P* > 0.9999; Kruskal–Wallis followed by Dunn’s multiple comparisons test]. Points represent mean ± SEM; bars indicate mean ± SEM.

**Fig. 2.**
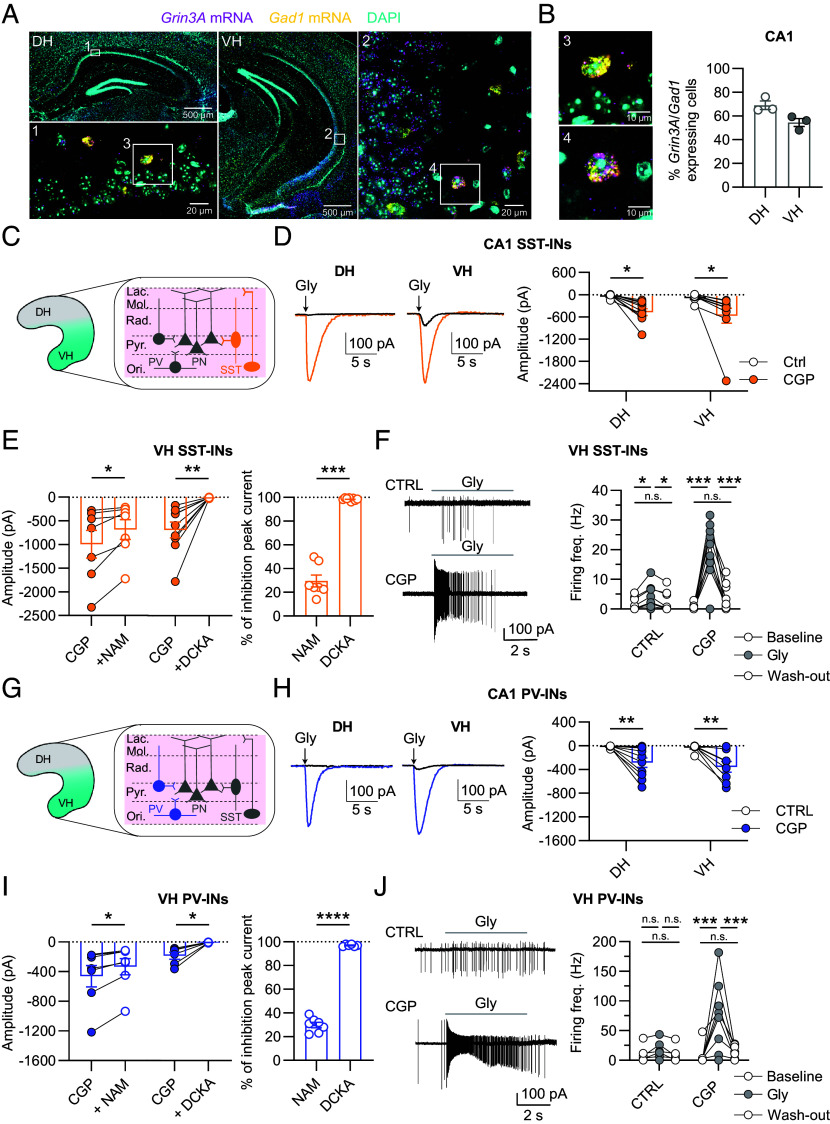
Hippocampal interneurons express eGlyRs in both dorsal and ventral subregions. (*A*) Mosaic images of the DH and VH labeled by RNAscope for *Grin3a* mRNA (magenta), *Gad1* mRNA (yellow), and DAPI (cyan). (1, 2) High-magnification images of the boxed areas in DH (1) and VH (2) CA1 hippocampal subregions. (*B*) *Left*: Higher-magnification images (3, 4) of the boxed areas in 1 and 2. *Right*: Quantification of the percentage of *Grin3a* mRNA positive cells in CA1 area of the DH and VH which also express the marker of inhibitory neurons *Gad1* (N = 3 animals per condition). (*C*) Schematic representation of hippocampal microcircuit with somatostatin positive interneurons (SST-INs) highlighted in orange. (*D*) Puffs of glycine (1 mM) in CGP (1 µM) induce inward currents (−65 mV) in SST-INs in both the VH and DH [VH (n = 10): CTRL vs CGP *P* = 0.0116; DH (n = 10): CTRL vs CGP *P* = 0.0262; two-way ANOVA followed by Sidak’s multiple comparisons test]. (*E*) Inhibition of glycine-induced currents (1 mM glycine, −65 mV) by NAM (30 µM) and DCKA (500 µM) [CGP vs CGP + NAM (n = 7), *P* = 0.0241; CGP vs CGP + DCKA (n = 9), *P* = 0.0034, Paired *t* test]. *Right*: Percentage of inhibition of the peak current [NAM (n = 7) vs DCKA (n = 9), *P* = 0.0002, Mann–Whitney test]. (*F*) Effect of glycine application (1 mM) on the firing of SST-INs of the VH with CGP and without CGP (CTRL). Loose cell-attached experiments [CTRL (n = 16): Baseline vs Gly *P* = 0.0471, Baseline vs Wash-out *P* = 0.3357, Gly vs Wash-out *P* = 0.0248; CGP (n = 11): Baseline vs Gly *P* = 0.0007, Baseline vs Wash-out *P* = 0.1128, Gly vs Wash-out 0.0007; two-way ANOVA followed by Tukey’s multiple comparisons test]. (*G*) Schematic representation of hippocampal microcircuit with parvalbumin positive interneurons (PV-INs) highlighted in violet. (*H*) Same as (*D*) but for PV-INs [VH (n = 8): CTRL vs CGP *P* = 0.0031; DH (n = 10): CTRL vs CGP *P* = 0.0061; two-way ANOVA followed by Sidak’s multiple comparisons test]. (*I*) Pharmacological inhibition of glycine-evoked currents (glycine 1 mM) by NAM (30 µM) and DCKA (500 µM) [CGP vs CGP + NAM (n = 7), *P* = 0.0156; CGP vs CGP + DCKA (n = 6), *P* = 0.0312, Wilcoxon test]. *Right*: percentage of inhibition of the peak current [NAM (n = 7) vs DCKA (n = 6), *P* < 0.0001, Unpaired *t* test]. (*J*) Same as in (*F*) but for PV-INs [CTRL (n = 10): Baseline vs Gly *P* = 0.1305, Baseline vs Wash-out *P* = 0.2750, Gly vs Wash-out *P* = 0.1174; CGP (n = 9): Baseline vs Gly *P* = 0.0206, Baseline vs Wash-out *P* = 0.4049, Gly vs Wash-out *P =* 0.0166; two-way ANOVA followed by Tukey’s multiple comparisons test]. Bars indicate mean ± SEM.

To characterize the expression of GluN3A subunit in the adult hippocampus with cellular resolution, we conducted multiplex FISH assays using RNAscope probes for the GluN3A subunit (*Grin3a*) and the vesicular glutamate transporter *vGlut1* (a marker of excitatory cells; Fig. 1*A*) or the GABA-synthesizing enzyme glutamate decarboxylase 1 *Gad1* (a marker of inhibitory interneurons; Fig. 2*A*). In the CA1 region of the DH, only a minority (14%) of pyramidal neurons (PNs) expressed *Grin3a* mRNA. In contrast, in the VH, the vast majority (91%) of PNs displayed strong *Grin3a* labeling (Fig. 1 *A* and *B*), thus confirming the graded expression of the *Grin3a* mRNA signal along the rostro-caudal axis of the adult hippocampus [also referred to as the dorsoventral or longitudinal or septotemporal axis (18, 20, 21)]. Analysis of protein expression on adult mouse hippocampi corroborated the dorsoventral *Grin3a* mRNA expression pattern (*SI Appendix*, Fig. S1*A*). PNs in the CA3 region and granule cells of the dentate gyrus (DG) lacked *Grin3a* mRNA signal (*SI Appendix*, Fig. S1*B*). Therefore, expression of *Grin3a* in PNs of the adult hippocampus is restricted to pyramidal cells of the CA1 layer with a strong enrichment in the ventral division.

We next investigated the presence of functional eGlyRs in the adult mouse hippocampus. We patch-clamped CA1 PNs and recorded responses to pressure applied glycine (1 to 10 mM) in the presence of a cocktail of inhibitors against most ionotropic glutamate, GABA, and (inhibitory) glycine receptors (Fig. 1*C*; see *Methods*). To maximize the detection of eGlyR-mediated currents, we also added to the bath solution the GluN1 antagonist CGP-78608 (CGP, 1 µM), that strongly reduces eGlyR desensitization (11, 15, 16). Glycine applications resulted in large inward currents in ventral CA1 PNs while no or tiny currents were evoked in dorsal CA1 PNs, unveiling a strong functional segregation of eGlyR expression in the VH vs DH (Fig. 1*D*). No currents were observed in ventral CA1 PNs when repeating the experiments in brain slices from GluN3A-KO animals (Fig. 1*D*), in agreement with the involvement of GluN1/GluN3A receptors. Pharmacological experiments using the nonselective eGlyR antagonist 5,7-Dichlorokynurenic acid (DCKA, 500 µM) (8, 9, 11) as well as the negative allosteric modulator EU1180–438 (NAM, 30 µM) (14) confirmed the presence of eGlyRs (Fig. 1*E*). In loose cell-attached recordings from VH CA1 PNs, glycine-puff applications in the presence of CGP induced intense and sustained spiking activity, thus confirming the depolarizing nature of eGlyR activity (*SI Appendix*, Fig. S1*B*). Since expression studies show marked cell specificity of GluN3A distribution (20, 21) (and see Fig. 1 *A* and *B* and *SI Appendix*, Fig. S1*A*), we further characterized functional expression of eGlyRs in principal cells of the VH. Whole-cell glycine-puffs experiments induced no current in both CA3 PNs and granule cells from the DG (Fig. 1*F*), fully matching the lack of *Grin3A* gene expression in these neuronal cell types.

In addition to forming glycine-gated GluN1/GluN3A eGlyRs, the GluN3A subunit has been proposed to assemble with both GluN1 and GluN2 subunits to form triheteromeric GluN1/GluN2/GluN3A receptors sensitive to both glutamate (and NMDA) and glycine as classical NMDARs but displaying reduced Mg^2+^ block (23–25). To evaluate the presence of GluN3A-containing triheteromeric receptors, current–voltage (I–V) relationships of puff-evoked NMDA currents (15) were examined in VH CA1 PNs (Fig. 1 *G* and *H*). As shown in Fig. 1*I*, I–V curves were not different between WT and GluN3A-KO animals. No difference was also observed between I–V curves from VH and DH CA1 PNs, these latter lacking GluN3A subunit (see above). Therefore, in the PNs of the adult hippocampus, GluN3A subunits are mainly if not exclusively associated with GluN1 to form diheteromeric GluN1/GluN3A eGlyRs.

### eGlyRs are Expressed in Interneurons from both Dorsal and VH.

To better understand the role of eGlyRs in controlling hippocampal circuits, we next characterized the presence and distribution of eGlyRs in interneurons (INs) of the adult hippocampus (Fig. 2*A*). Multiplex FISH imaging revealed that an important fraction of INs expressed *Grin3a* mRNA in both the dorsal and ventral CA1 region (Fig. 2*B*). Labeling for *Grin3a* was also observed in INs of the CA3 and DG regions of both DH and VH (*SI Appendix*, Fig. S2*A*). Glycine puff experiments on SST-INs revealed large glycine-gated inward currents in the ventral but also DH (Fig. 2 *C* and *D*), in line with our expression data. These glycine-evoked currents displayed similar pharmacology to glycine-evoked currents from VH CA1 PNs, with partial and full inhibition by the compounds NAM (30 µM) and DCKA (500 µM), respectively (Fig. 2*E* and *SI Appendix*, Fig. S2*B*). Similar to VH CA1 PNs, a massive increase in the spiking activity of VH SST INs was observed in loose cell-attached recordings upon glycine application in the presence of CGP-78608 (Fig. 2*F*). Notably, puffs of glycine alone were sufficient to enhance SST-IN discharge, although at lower firing rates than in the presence of CGP-78608 (Fig. 2*F*). Repeating this set of experiments in parvalbumin positive interneurons (PV-INs) unveiled glycine-evoked excitatory inward currents in both the VH and DH (Fig. 2 *G*–*J* and *SI Appendix*, Fig. S2*C*). Overall, these data show that eGlyRs are highly expressed in both dorsal and ventral hippocampal SST-INs and PV-INs where they control cell excitability.

### eGlyRs are Tonically Activated.

**Fig. 3.**
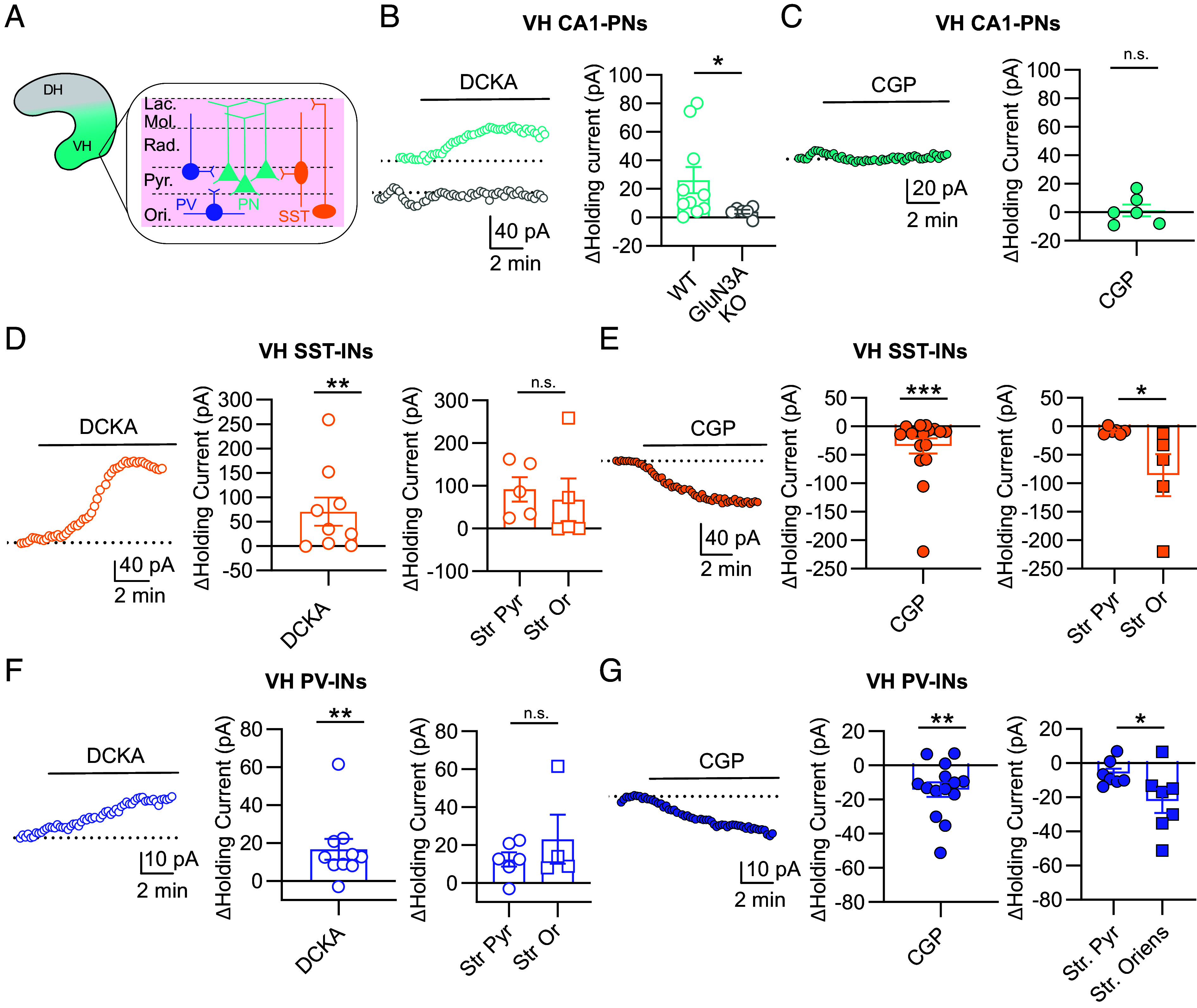
Tonic activation of eGlyRs. (*A*) Schematic representation of hippocampal microcircuit with PNs in light blue, SST-INs in orange and PV-INs in violet. (*B*) Effect of DCKA (500 µM) on holding currents in WT and GluN3A-KO CA1 PNs of the VH [WT (n = 10) vs GluN3A-KO (n = 6), *P* = 0.0420, Mann–Whitney test]. (*C*) Effect of CGP (1 µM) on holding currents in CA1 PNs of the VH [CGP (n = 6) *P* > 0.9999, One-sample Wilcoxon test]. (*D*) Effect of DCKA (500 µM) on holding currents in SST-INs of the VH [DCKA (n = 9), *P* = 0.0078, one-sample Wilcoxon test]. *Right*: Similar effects between the *Stratum Pyramidale (Str Pyr)* and the *Stratum Oriens (Str Or)* [*Str Pyr* (n = 5) vs *Str Or* (n = 5), *P* = 0.3095, Mann–Whitney test). (*E*) Effect of CGP (1 µM) on holding currents in SST-INs of the VH [CGP (n = 17), *P* = 0.0002, one-sample Wilcoxon test]. *Right*: Larger effect in the *Str Or* compared to the *Str Pyr* [*Str Pyr* (n = 6) vs *Str Or* (n = 6), *P* = 0.0173, Mann–Whitney test]. (*F*) As in (*D*) but for PV-INs [DCKA (n = 10), *P* = 0.0039, one-sample Wilcoxon test]. *Right*: Similar effects between *Str Pyr* and *Str Or* [*Str. Pyr* (n = 6) vs *Str. Or* (n = 4), *P* = 0.9143, Mann–Whitney test]. (*G*) As (*E*) but for PV-INs of the VH [CGP (n = 14), *P* = 0.0023, One-sample Wilcoxon test]. As for SST-INs, CGP-induced holding currents are larger in the *Str Or* than in the *Str Pyr* [*Str Pyr* (n = 7) vs *Str Or* (n = 7), *P* = 0.0262, Mann–Whitney test]. Bars indicate mean ± SEM.

We next assessed the mode of activation of eGlyRs in the VH. Given the scarcity of glycinergic innervation in the hippocampus [and in the forebrain in general (26)] and the relatively high affinity for glycine of the activating subunit GluN3A (8–11, 27), an enticing hypothesis is that eGlyRs operate as sensors of extracellular ambient glycine levels, as previously proposed in other brain structures (16). Accordingly, we tested the effect of GluN1/GluN3A receptor inhibition on holding currents of different cell types of the VH (Fig. 3*A*). DCKA application reduced holding currents in CA1 PNs from WT but not GluN3A-KO slices (Fig. 3*B*), showing that eGlyRs can generate sustained inward currents. Preventing eGlyRs desensitization with CGP-78608 had no significant effect on holding currents (Fig. 3*C*), suggesting that extracellular glycine concentrations around CA1 PNs are not sufficient to occupy eGlyR GluN1 sites (auto-inhibitory desensitizing sites) (16). DCKA also reduced holding currents from SST-INs of the VH (Fig. 3*D*) but surprisingly not of the DH (*SI Appendix*, Fig. S3*A*). CGP application increased inward holding currents of SST-INs neurons from both ventral and dorsal regions (Fig. 3*E* and *SI Appendix*, Fig. S3*B*), revealing a difference in eGlyR GluN1 site occupancy between SST-INs and PNs. Remarkably, when separating SST-INs located in the *stratum oriens* vs *stratum pyramidale*, a clear difference in CGP-78608 sensitivity emerged. Only SST-INs with their cell bodies in the *stratum oriens* displayed an enhancement of tonic currents by CGP-78608 (Fig. 3*E*). The minimal CGP-78608 sensitivity of tonic currents from SST-INs in the *stratum pyramidale* could not be accounted by a specific scarcity of eGlyRs in these cells (similar extent of DCKA inhibition between the two layers; Fig. 3*D*). Repeating these experiments and analyses in VH PV-INs revealed a similar pattern, with a group of PV-INs in the *stratum oriens* displaying eGlyR-mediated tonic currents strongly sensitive to CGP-78608 and a group of CGP-78608 insensitive PV-INs located in the *stratum pyramidale* (Fig. 3*G*). As for SST-INs, the extent of DCKA inhibition was similar between the two layers (Fig. 3*F*). Altogether, these results establish that eGlyRs are usually tonically active in cells where they are expressed, thus participating in holding currents. They also unveil unsuspected differences in eGlyR GluN1 site occupancy between hippocampal layers, suggesting that local ambient glycine concentrations might differ depending on the cellular microenvironment (see Discussion).

### eGlyRs Account for the Reduced VH LTP and Its Regulation by Corticosterone.

**Fig. 4.**
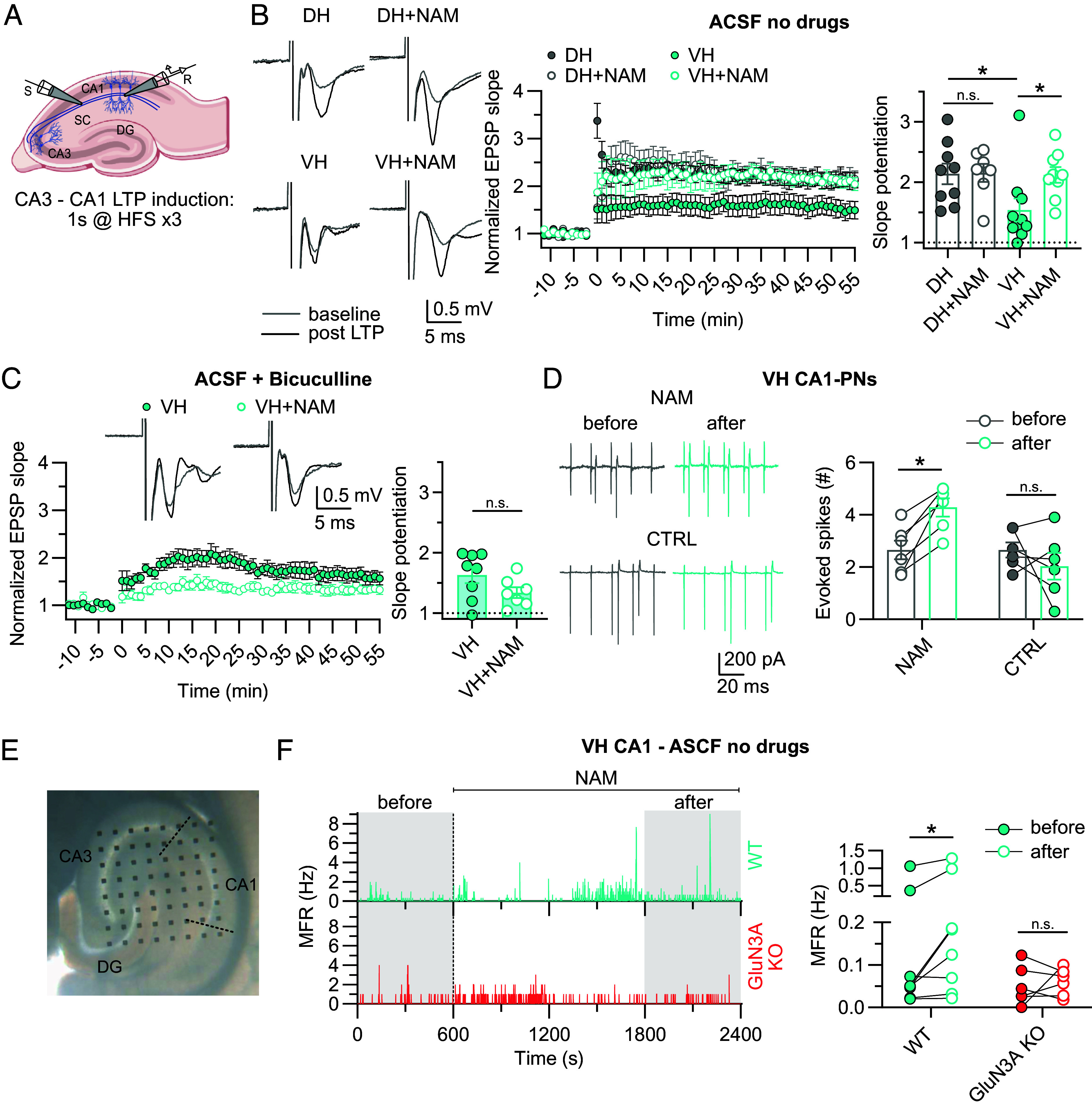
eGlyRs control LTP in the VH. (*A*) Schematic representation of the CA3-CA1 LTP induction protocol in the hippocampus. (*B*) NAM (30 µM) potentiates LTP in the VH but not in the DH [DH (n = 9) vs DH+NAM (n = 7), *P* > 0.9999; VH (n = 10) vs VH+NAM (n = 9), *P* = 0.0440, DH vs VH, *P* = 0.0338. Kruskal–Wallis test followed by Dunn’s multiple comparisons test]. (*C*) NAM (30 µM) does not potentiate VH LTP in the presence of bicuculline (10 µM) [VH (n = 8) vs VH+NAM (n = 7), *P* = 0.1077, Unpaired *t* test]. (*D*) NAM (30 µM) enhances the firing of CA1 PNs of the VH in response to extracellular stimulation [NAM (n = 6): before vs after, *P* = 0.0138; CTRL (n = 6) before vs after, *P* = 0.4071; two-way ANOVA followed by Sidak’s multiple comparisons test]. (*E*) Multielectrode array positioned on a hippocampal slice (VH). The dotted lines delimit the CA1 area. (*F*) *Left*: Representative time course of CA1 spiking activity in WT and GluN3A-KO slices. *Right*: Mean firing rate (MFR) is increased upon NAM (30 µM) application in WT but not GluN3A-KO CA1 [WT (n = 8): before vs after NAM, *P* = 0.0442; GluN3A-KO (n = 6) before vs after NAM, *P* = 0.9888; two-way ANOVA followed by Sidak’s multiple comparisons test]. Bars indicate mean ± SEM. In the representative traces of LTP experiments, the stimulus artifact was cut.

Intrigued by the differential expression pattern of eGlyRs in the dorsal and VH, we wondered whether eGlyRs contribute to functional features that distinguish the two subregions. Among these features, the magnitude of CA3-CA1 long-term potentiation (LTP) has been shown to vary along the hippocampal longitudinal axis, the extent of LTP being substantially weaker in the VH than in the DH (28–31). We tested the hypothesis that the strong enrichment of eGlyRs in the VH participates in the reduced LTP. Field potential recordings combined with high frequency stimulation of the Shaffer Collaterals confirmed a significantly higher magnitude of LTP in the DH compared to the VH (Fig. 4 *A* and *B*). Critically, acute inhibition of eGlyRs with NAM enhanced LTP in the VH but had no effect in the DH (Fig. 4*B*). Thus, eGlyRs play an active role in controlling LTP magnitude in the VH. We investigated the mechanism by which the inhibition of an excitatory conductance (eGlyRs) enhances, rather than decreases, LTP. Since NAM application dampens eGlyR activity in both excitatory and inhibitory neurons (Figs. 1 and 2), we investigated NAM effect on VH LTP in the absence of the inhibitory network to assess the contribution of INs to the eGlyR-mediated regulation of LTP. We thus repeated LTP experiments in the VH in the presence of the competitive GABA_A_ receptor antagonist bicuculline (10 µM) to block the inhibitory component of the network. In these conditions, NAM did not significantly affect LTP (Fig. 4*C*), revealing the key role of eGlyRs on INs in the control of VH LTP. We obtained further evidence of the critical influence of IN eGlyRs on the VH neuronal network by measuring CA1 PN spiking activity in cell-attached recordings with both excitatory and inhibitory neurons unperturbed (no bicuculline). In such conditions, acute NAM application increased the spiking activity induced by short bursts of synaptic stimulation (Fig. 4*D*), indicating an increased excitability of PNs. Conversely, in the DH, and in line with LTP experiments, NAM application did not affect PNs excitability upon synaptic stimulation (*SI Appendix*, Fig. S4*A*). To go deeper in the underlying mechanism, we assessed the contribution of eGlyRs in SST-INs, these interneurons being particularly enriched in eGlyRs (Fig. 2). Downregulation of GluN3A selectively in VH SST-INs (*SI Appendix*, Fig. S4*B*) tended to increase CA3-CA1 LTP amplitude, similarly to the NAM, although the effect did not reach statistical significance (*SI Appendix*, Fig. S4*C*). To get a broader view of hippocampal network activity, we took advantage of multielectrode array (MEA) experiments (Fig. 4*E*) and found that CA1 spiking activity was significantly augmented upon NAM application in WT but not GluN3A-KO mice (Fig. 4*F*). Overall, these results indicate that eGlyRs directly control the magnitude of LTP through modulation of network excitability in the adult VH.

**Fig. 5.**
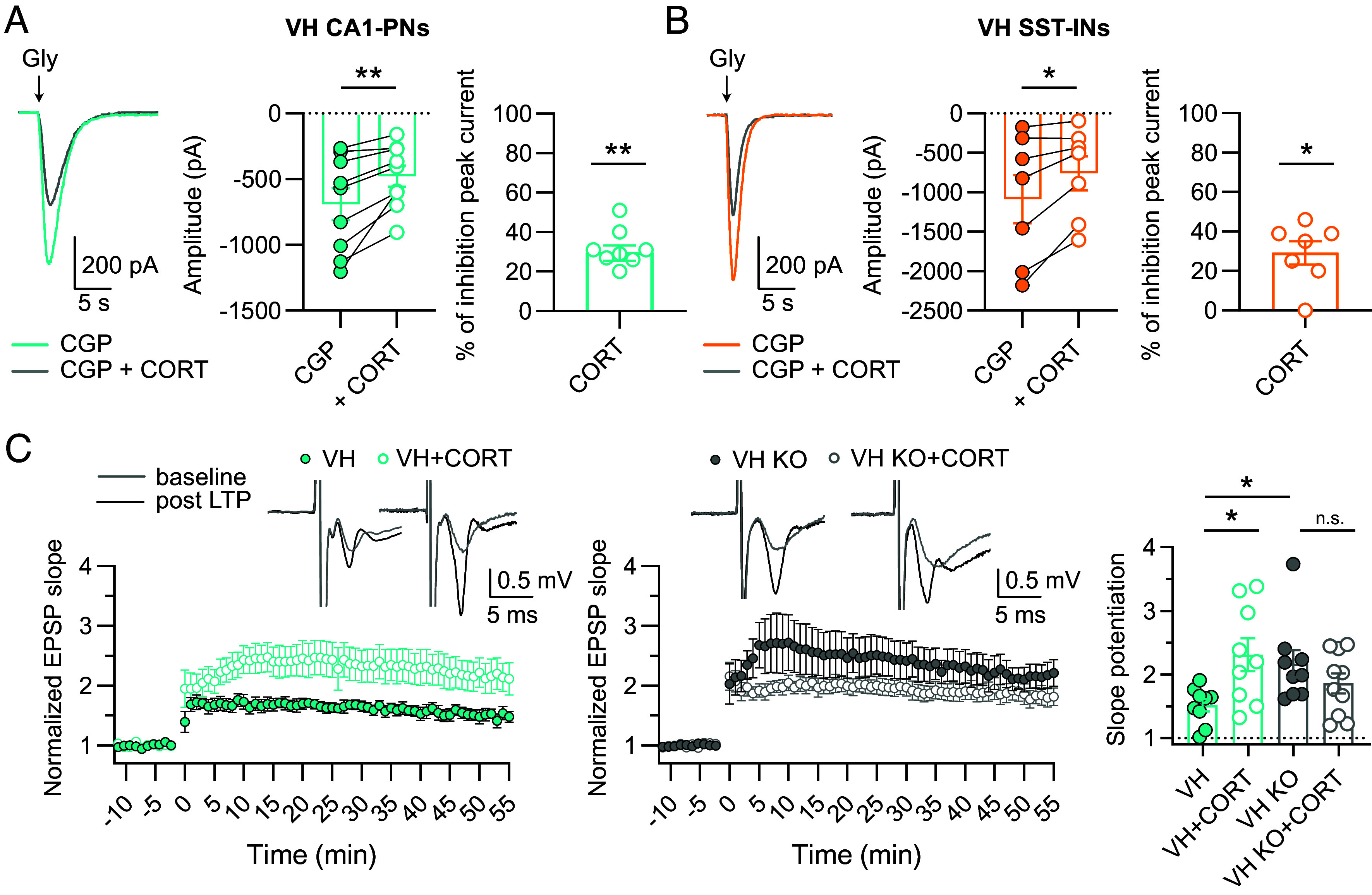
Corticosterone potentiates LTP in the VH by inhibiting eGlyRs. (*A*) Pharmacological inhibition by corticosterone (CORT, 1 µM) of glycine-evoked currents (glycine 1 mM, −65 mV) in the presence of CGP (1 µM) in CA1 PNs of the VH [CGP vs CGP + CORT (n = 9), *P* = 0.0058, Paired *t* test]. *Right*: Percentage of inhibition of the peak current by CORT [CORT (n = 9), *P* = 0.0039, one-sample Wilcoxon test]. (*B*) Inhibition by CORT (1 µM) of glycine-induced currents in CA1 SST-INs of the VH [CGP vs CGP + CORT (n = 7), *P* = 0.0208, Paired *t* test]. *Right*: Percentage of inhibition of the peak current [CORT (n = 7), *P* = 0.0312, one-sample Wilcoxon test]. (*C*) CORT (1 µM) application enhances LTP in the VH of WT but not GluN3A-KO mice [VH (n = 9) vs VH+CORT (n = 9), *P* = 0.0253; VH vs VH KO (n = 9), *P* = 0.0392, VH KO vs VH KO + CORT (n = 10), *P* > 0.9999. Kruskal–Wallis test followed by Dunn’s multiple comparisons test]. Bars indicate mean ± SEM. In the representative traces, the stimulus artifact was cut.

Another property that distinguishes the VH from the DH is their sensitivity to the stress-hormone corticosterone. While corticosterone inhibits CA3-CA1 LTP in the DH, it potentiates LTP in the VH (28). We hypothesized that corticosterone may act as an endogenous modulator of VH LTP via eGlyRs, mimicking the NAM effect. Acute corticosterone (1 µM) applications inhibited eGlyR-mediated currents, an effect observed in both VH PNs and VH SST-INs (Fig. 5 *A* and *B*). Coherently, corticosterone prevented the glycine-mediated enhancement of VH-SST IN firing (*SI Appendix*, Fig. S5*A*). We then performed CA3-CA1 LTP experiments in VH slices of WT and GluN3A-KO mice. The extent of LTP was markedly enhanced by corticosterone (1 µM) incubation in WT slices (Fig. 5*C*). On the opposite, corticosterone had no effect on LTP in the VH of GluN3A-KO mice (Fig. 5*C*), evidencing that the potentiation of LTP by corticosterone requires eGlyR expression. We also noticed that the magnitude of LTP in the VH of GluN3A-KO mice was larger compared to WT animals (Fig. 5*C*; see also ref. 22), an effect mimicking acute eGlyR inhibition by NAM in WT slices (see Fig. 4*B*). To exclude any ceiling effects in GluN3A-KO slices, we also tested a submaximal LTP induction protocol (1s HFS instead of 3× 1s HFS) and found again no effect of corticosterone 1 µM (*SI Appendix*, Fig. S5*B*), confirming that corticosterone potentiation of VH LTP needs functional eGlyRs. In line with LTP data, corticosterone 1 µM increased the spiking activity induced by short bursts of synaptic stimulation (*SI Appendix*, Fig. S5*C*), as previously observed with the NAM (Fig. 4*D*). These findings reveal that eGlyRs are essential regulators of synaptic plasticity in the VH and mediate the neuromodulatory action of corticosterone on VH LTP.

### VH eGlyRs Regulate Anxiety-Related Behaviors.

**Fig. 6.**
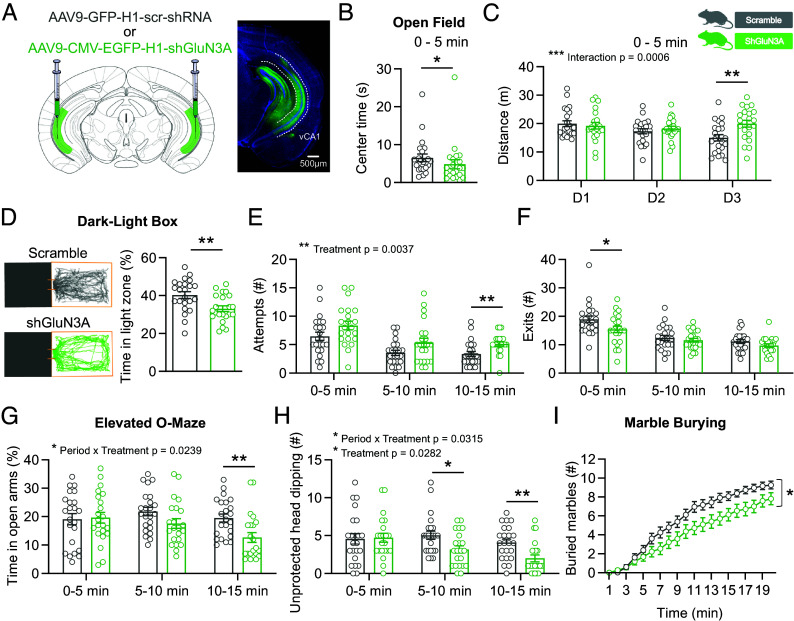
eGlyRs in the VH contribute to anxiety-related behaviors. (*A*) *Left*: Sites in the VH used for bilateral viral injections of either AAV9-GFP-H1-scr-shRNA (scramble) or AAV9CMV-EGFP-H1-shGluN3A (shGluN3A). *Right*: Infected neurons expressing EGFP in the ventral CA1 region (vCA1). (*B* and *C*) Open-field test (OF). (*B*) ShGluN3A-treated mice treated spent less time in the center of the OF during the first 5 min than control animals (scramble) (*P* = 0.0374, Mann–Whitney test). (*C*) Distance traveled from day 1 (D1) to day 3 (D3) during the first 5 min [Day F(2, 86) = 5.063, *P* = 0.0092; Treatment F(1, 43) = 2.106; Day × Treatment F(2, 86) = 8.088, *P* = 0.0006; D3: shGluN3A vs scramble *P* = 0.0038]. (*D–F*) Dark–light test (DL). (*D*) ShGluN3A-treated mice spent less time in the light area than control animals (*P* = 0.0030, Mann–Whitney test). (*E*) Number of exit attempts [Period F(2, 86) = 21.62, *P* < 0.0001; Treatment F(1, 43) = 9.426, *P* = 0.0037; Period × Treatment F(2, 86) = 0.005, *P* = 0.9951; 10 to 15 min: shGluN3A3a vs scramble *P* = 0.0042]. (*F*) Number of exits during the first 5 min [Period F(2, 86) = 48.13, *P* < 0.0001; Treatment F(1, 43) = 4.061, *P* = 0.0502; Period × Treatment F(2, 86) = 1.941, *P* = 0.1498; 0 to 5 min: shGrin3a vs scramble *P* = 0.0377]. (*G* and *H*) Elevated O-maze test. (*G*) ShGluN3A-treated mice spent less time in the open arms during the time period 10 to 15 min than control animals [Period F(2, 86) = 4.396, *P* = 0.0154; Treatment F(1, 43) = 3.611, *P* = 0.0641; Period × Treatment F(2, 86) = 3.899, *P* = 0.0239; 10 to 15 min: shGluN3A vs scramble *P* = 0.0039]. (*H*) Number of unprotected head dipping [Period F(2, 86) = 6.256, *P* = 0.0033; Treatment F(1, 43) = 5.156, *P* = 0.0282; Period × Treatment F(2, 86) = 3.602, *P* = 0.0315; 5 to 10 min: shGluN3A vs scramble *P* = 0.0136; 10 to 15 min: shGluN3A vs scramble *P* = 0.0022]. (*I*) Marble burying test. ShGluN3A-treated animals buried marbles at a slower pace than control animals [Time F(19, 817) = 192.3, *P* < 0.0001; Treatment F(1, 43) = 5.087, *P* = 0.0292; Time × Treatment F(19, 817) = 3.589, *P* < 0.0001]. Points represent mean ± SEM; Scramble N = 23 vs ShGluN3A N = 22 for each test; bars indicate mean ± SEM. Except where otherwise specified, a mixed effect restricted maximum likelihood (REML) model has been used followed by Sidak’s multiple comparison test.

Considering the well-established involvement of the VH in emotional processing (32–36), we next investigated the role of VH eGlyRs in anxiety-related behaviors. For that purpose, we performed a battery of commonly used tests assessing approach-avoidance conflict (*SI Appendix*, Fig. S6*A*) combined to a shRNA-based gene silencing approach (16, 37) (GluN3A shRNA) which efficiently reduced eGlyR activity in the VH (>95% inhibition of glycine puff currents compared to control scramble shRNA; Fig. 6*A* and *SI Appendix*, Fig. S6*B*). Downregulation of *Grin3A* in the VH did not induce changes in basal locomotor activity, as assessed in the open field test in day one and over a 3-day period (*SI Appendix*, Fig. S6 *C* and *E*). In contrast, analysis of the time spent in the center of the open field during the first 5 min—the most anxiogenic location and time interval for the animals—revealed a significant reduction in the GluN3A shRNA group compared to the scramble one (Fig. 6*B*). Moreover, analysis of the total distance traveled during the subsequent days showed that on day 3, the distance traveled was greater in GluN3A *vs* scramble shRNA (Fig. 6*C* and *SI Appendix*, Fig. S6*D*), highlighting a decreased habituation to the open field in the GluN3A shRNA animals. In the dark–light test (DL), GluN3A shRNA treated mice spent less time in the anxiogenic light area compared to controls (Fig. 6*D* and *SI Appendix*, Fig. S6*F*). Additionally, the number of attempts to exit the dark compartment increased (Fig. 6*E*), but the number of full exits from this compartment decreased (first 5 min; Fig. 6*F*). Significant effects of treatment were also observed when considering the time spent immobile in the light compartment (*SI Appendix*, Fig. S6*G*). Altogether, these results indicate increased anxiety in GluN3A shRNA mice. To investigate further the role of VH GluN3A-containing receptors in anxiety-related behavior, we tested the animals in the elevated O-maze, where the time spent in open *vs* closed arms correlates with anxiety levels. The percentage of time spent in the open arms revealed a decreased exploration over the time in the GluN3A shRNA group compared to the control group (10 to 15 min; Fig. 6*G*). The number of unprotected head dips also decreased in the GluN3A shRNA treated animals (Fig. 6*H*), revealing heightened anxiety with reduced risk-taking actions. Finally, animals were tested for active avoidance of a neutral stimulus using the marble burying test. GluN3A shRNA and controls showed the same latency to the first buried marble and the same total number of buried marbles in the 20 min test (*SI Appendix*, Fig. S6 *H* and *I*), yet the pace at which animals buried the marbles differed between the two groups (Fig. 6*I*). This latter phenotype suggests that shGluN3A animals need more time to start actively influencing their environment due to heightened anxiety. Taken together, the results of the behavioral tests show that downregulation of GluN3A results in a general increase in anxiety in different environmental contexts. This supports an important role of VH eGlyRs in the regulation of emotionality and adaptive behaviors.

## Discussion

In this work, we uncover that GluN1/GluN3A eGlyRs, a recently identified new type of neurotransmitter receptor, form a genuine and biologically relevant signaling modality in the mature hippocampus. Combining anatomical, physiological, and behavioral data, we show that hippocampal eGlyRs are functional and strongly enriched in the ventral division of the adult hippocampus, due to a marked dorsoventral gradient of expression in the CA1 pyramidal cells. We also show that hippocampal eGlyRs control neuronal excitability via volume transmission. Despite the presence of eGlyRs in CA1 PNs, the overall impact of eGlyR on the VH network activity is dominated by eGlyR function in interneurons. Thus, eGlyRs account for the dampened LTP in the VH compared to the DH, a difference known for decades but unexplained up to now. We also demonstrate that eGlyRs are required for the stress hormone corticosterone to regulate the extent of synaptic plasticity in the VH, pointing to eGlyRs as mediators of the neuroendocrine stress response. In line with this, we provide evidence that eGlyRs in the VH contribute to the modulation of anxiety-related behaviors. Overall, our data establish eGlyR signaling as an important functionality that provides unique attributes to the VH. Because the VH relates to emotion, stress, and affect, the discovery of functional eGlyRs in this structure has important consequences for our understanding of brain function, behavior, and drug development.

Our data highlight the strong cell type specificity of GluN3A expression but also the strict match between the expression of *Grin3a* mRNA and the presence of operational eGlyRs. Among hippocampal PNs, we found a clear exclusion of GluN3A (and of eGlyRs) from adult CA3 PNs and DG granule cells, while adult CA1 PNs showed a marked dichotomy between dorsal cells, with little *Grin3a* mRNA and eGlyR expression, and ventral cells, highly enriched in *Grin3a* mRNA and eGlyRs. This dichotomy results from a specific developmental downregulation of *Grin3a* expression in dorsal CA1 PNs since juvenile CA1 PNs in the DH express high levels of GluN3A(21) which also translate into eGlyR expression (11, 13, 14). We also observed GluN3A expression and functional eGlyRs in both adult DH and VH PV-INs and SST-INs, with particularly high signals in the latter cell types. In fact, *Grin3a* is considered as a secondary marker of SST-INs in the forebrain (1, 21, 38), in line with our current and previous observations that eGlyRs are expressed at particularly high density in these cells [Fig. 2*D*; and see ref. 16]. Accordingly, application of glycine alone (i.e. without CGP-78608) is sufficient to trigger inward currents in SST-INs and boost their spiking activity (Fig. 2 *D* and *F*). Conversely, we observed that not all PV-INs of the hippocampus express functional eGlyRs (Fig. 2*H*), in line with single-cell transcriptomic data (https://portal.brain-map.org/atlases-and-data/rnaseq). In general, we notice a perfect match between the expression of GluN3A within a neuronal type and the presence of functional eGlyRs, be it in the medial habenula (15), the basolateral amygdala, and the somatosensory cortex (16), and now the hippocampus. Notably, the presence of functional triheteromeric GluN1/GluN2/GluN3A could not be detected in any of these regions, supporting diheteromeric GluN1/GluN3A receptors as the main population of GluN3A-containing NMDARs in the adult brain.

Similar to other forebrain regions, the hippocampus receives little to no glycinergic innervation (26, 39), indicating that eGlyRs are unlikely to be activated in a phasic manner by synaptically released glycine. Rather, we show that eGlyRs mediate tonically active inward currents in hippocampal cells (PNs, SST-Ins, and PV-INs) in which they are expressed. Extrapolating from previous findings (16), we hypothesize that hippocampal eGlyRs act as sensors of ambient extracellular glycine known to be present in the extracellular space (40, 41) (see also ref. 16). Interestingly, we reveal differences in the occupancy of the eGlyR glycine sites depending on the location of the neurons in the hippocampal layers. Indeed, we show that CGP-78608 potentiates tonic eGlyR currents in the *stratum oriens* but not in the *stratum pyramidale*. Differences in ambient glycine levels between subregions of the hippocampus provide a possible explanation of this effect. However, we cannot rule out the possibility of differences in the receptor’s intrinsic properties such as affinity for glycine, although the fact that CGP-78608 sensitivity varies between hippocampal layers regardless of cell type (in PNs and INs) supports differences in local ambient glycine concentrations. With its ability to control the local glycine availability in the forebrain through glycine uptake or release [reverse transport mode; (42, 43)], the glycine transporter GlyT1 appears strategically positioned to influence eGlyR activity. In the hippocampus, GlyT1 is expressed by astrocytes and neurons with differences in subregions and layers which may account for diverse glycine microenvironments (39, 44, 45). In particular, GlyT1 appears denser around the cell bodies in all pyramidal layers (39, 44). This nicely echoes our observation (current study) that eGlyR glycine site occupancy is lower in the stratum pyramidale compared to the *stratum oriens* (Fig. 3 *E* and *G*). Moreover, astrocytes in the VH are more complex and ramified than in the corresponding layers of the DH (46). Upon high-frequency stimulation of CA3–CA1 Shaffer collateral synapses, extracellular glycine transiently increases (47). Neuromodulators, such as dopamine, can also modify glycine levels through regulation of astrocytic GlyT1 (41). All these observations lend support to ambient extracellular glycine being dynamically regulated and varying according to subregions, with an important role of astrocytic GlyT1. Because eGlyR activity is directly controlled by glycine availability, a better understanding of the regional and temporal variations in glycine levels is needed.

Our data also establish that GluN3A and corresponding eGlyRs confer distinct properties to the VH thus contributing to the unique role of the VH in brain function and behavior. The existence of a deep anatomical and functional differentiation across the longitudinal axis of the hippocampus is well documented (35, 36). The dorsal subregion performs primarily cognitive function including memory formation and spatial learning, while the ventral subregion is mainly involved in emotions and anxiety (32, 33, 48). Coherent with this functional segmentation, the extrinsic connectivity differs between the two subregions with the dorsal part of the hippocampus receiving major visuospatial and other sensory inputs from the cortex and the ventral part communicating primarily with subcortical structures such as the amygdala and the hypothalamus (32, 33, 48, 49). Molecular and physiological evidence also indicate that the dorsal and ventral zones of the hippocampus form distinct functional domains (18, 50), although the relation between regionally-enriched gene expression and specific network properties remains ill defined. Previous observations (18, 21) have shown that *Grin3a* belongs to a pool of genes differentially expressed along the dorsoventral axis of the hippocampal CA1 layer, with a strong enrichment in the ventral zone. Our FISH experiments fully confirm this preferential expression of GluN3A in VH CA1 PNs, yet show that INs do not follow the same segregation rule, expressing GluN3A in both the DH and VH. Our electrophysiological analysis also reveals that this pattern of GluN3A expression directly translates into an overlapping map of functional eGlyR expression, endowing distinct functionalities to neurons and circuits. CA1 PNs from the VH are more excitable and have a more depolarized resting membrane potential than DH CA1 PNs (50, 51). With their ability to mediate persistent inward currents (Fig. 3 *B*-*G*), we propose that eGlyRs are responsible, at least partially, of these differences. The VH and DH also strikingly differ in their ability to undergo LTP (28–31). Our current work shows that eGlyRs play a key role in this differentiation, acting as a brake for LTP in the VH. GluN3A-KO mice are known to display increased LTP (22, 24, 52) but developmental effects on network architecture render interpretation of this phenotype difficult. Here, we unveil that, in the mature VH, acute and specific inhibition of eGlyRs by NAM increases the excitability of CA1 PNs and of CA1 neuronal network in general, resulting in enhanced LTP. That the inhibition of an excitatory conductance (eGlyRs) on PNs enhances, rather than decreases, LTP could appear counterintuitive. However, in the absence of the inhibitory network (i.e., in bicuculline), NAM loses its potentiating effect revealing the critical role of IN eGlyRs in regulating VH LTP. Although eGlyRs are expressed in INs of both DH and VH, differences in local interneuron density and connectivity between the two subregions (53–55) likely account for the lack of NAM effect on DH LTP. In particular, the number of SST-INs, which highly express eGlyRs (see Fig. 2), is higher in the VH compared to the DH (55). The striking enrichment of eGlyRs in VH CA1 PNs is likely to be another contributing factor for the differential NAM effect on VH *vs* DH LTP and on local network excitability (via PN–PN and PN–IN connections). By inhibiting eGlyRs from various cell types in the VH (CA1 PNs, SST–INs, PV–INs), NAM likely produces an overall disinhibitory effect of CA1 PNs, resulting in increased LTP.

The sensitivity of hippocampal synaptic plasticity to stressors is another parameter that distinguishes the VH from the DH. In particular, the endogenous stress hormone corticosterone has been reported to potentiate LTP in the VH but not in the DH, an effect involving the activation of mineralocorticoid receptors (28). Our results demonstrate that the expression of eGlyRs is required for the regulation of LTP in the VH by corticosterone. Specifically, we show that eGlyRs are a downstream target of corticosterone action, resulting in eGlyR inhibition, increased excitability of CA1 PNs and LTP enhancement, mimicking the pharmacological effect of the NAM compound. Our results are consistent with the finding that corticosterone increases the excitability of CA1 PNs and reduces the frequency but not the amplitude of inhibitory postsynaptic currents exclusively in the VH (56). Given the relatively rapid effect of corticosterone on eGlyRs (min time scale; Fig. 5 *A* and *B*), we envision a nongenomic mechanism involving mineralocorticoid and/or glucocorticoid receptors (57). Further investigations are now needed to dissect the signaling pathways linking corticosterone, its receptors, and the regulation of eGlyR activity.

Finally, our in vivo experiments in mice reveal that eGlyRs in the VH are physiologically relevant by participating in anxiety-related behaviors. Previous studies based on GluN3A full knockout and GluN3A selective deletion from all excitatory neurons during adulthood indicate enhanced learning and memory abilities (19, 52). Here, we focused on well-established behaviors which depend primarily on VH and not DH function (32, 34). We show that the selective downregulation of eGlyRs in the VH overall leads to increased anxiety. This contrasts with the anxiolytic effects of conventional NMDAR antagonists such as MK-801, ifenprodil, and APV, administered systematically or locally in the VH (58, 59). In fact, GluN1/GluN3A eGlyRs and conventional GluN1/GluN2 NMDARs, although phylogenetically and structurally related (2, 4, 5, 60), appear highly divergent in their distribution, mechanism, and role. GluN1/GluN2 receptors are ubiquitously expressed and operate under a phasic regime at glutamatergic synapses where they act as coincidence detectors (2, 3). GluN1/GluN3A receptors on the other end are expressed in discrete brain regions and do not cluster at synapses but operate under a tonic regime to produce glycine-mediated neuromodulation (this work and see ref. 1). These diverging properties between eGlyRs and conventional NMDARs might turn into an advantage for possible future GluN3A-targeted therapeutics that might escape the poor tolerability and abuse liability associated with many GluN1/GluN2 targeting drugs (61, 62). Based on the current and previous studies (15, 16), a pattern emerges whereby GluN3A and eGlyRs are highly enriched in a set of interconnected brain regions regulating emotional and motivational states: the medial habenula, the lateral amygdala, and the VH. We note also that GluN3A is strongly expressed in the hypothalamus (1), a key hub for internal state control. In humans, *GRIN3A* displays a distribution pattern highly similar to that of rodents (1), and alterations in *GRIN3A* expression or sequence are associated with several neuropsychiatric conditions such as schizophrenia, autism, epilepsy, and addiction (1, 63–65). Increasing evidence also links glycine dyshomeostasis to CNS disorders including schizophrenia, autism spectrum disorder, and glycine encephalopathy, a genetic disease associated with brain malformations and severe neurological symptoms (42, 66–68). This strong link between glycine dyshomeostasis and CNS disorders highlights the importance of clarifying the role and dynamic of brain glycine signaling. Our findings of functional, cell-specific eGlyRs that confer unique properties to VH circuits and associated behaviors provide a significant contribution in this direction.

## Methods

C57BL/6J, GluN3A-KO and WT littermates, Sst-IRES-Cre X Ai9 (RCL-tdT), and PV-Cre X Ai9 (RCL-tdT) mice (2 to 3.5 mo old, both males and females) were used in this study. Mice were maintained in laboratory cages under a standard 12 h light/dark cycle with food and water ad libitum. All experiments were performed in compliance with the French and European regulations (EU Directive 2010/63, French Law 2013-118, February 6th, 2013, authorization numbers #28867 and #44478). Experimental procedures including brain slice preparation, ex vivo electrophysiology, multielectrode array (MEA) recordings, RNAscope In Situ Hybridization, viral injection, and behavioral experiments were detailed in *SI Appendix*, *Supplementary Methods*. Data analysis and statistics are also included in *SI Appendix*, *Supplementary Methods*.

## Supplementary Material

Appendix 01 (PDF)

## Data Availability

Excel file data have been deposited in Mendely Data (https://doi.org/10.17632/bgp5zxcxgz.1) (69).
