# Supplementary material for: Excitatory glycine receptors control ventral hippocampus synaptic plasticity and anxiety-related behaviors
Source: Proc Natl Acad Sci U S A. 2025 Sep 9;122(37):e2501118122. doi: 10.1073/pnas.2501118122 (PMC12452925; doi:10.1073/pnas.2501118122)
Supplement: Supplementary file 1 — Appendix 01 (PDF) [file pnas.2501118122.sapp.pdf]

Supporting Information for

Excitatory glycine receptors control ventral hippocampus synaptic plasticity and anxiety-related behaviors

Lara Pizzamiglio[a], Elise Morice[b], Cécile Cardoso[a], Simon Bossi[a], Caroline Mailhes-Hamon[a], Moritz von Heimendahl[c], Gabrielle Girardeau[b] and Pierre Paoletti[a,*]

[a] Institut de Biologie de l'Ecole Normale Supérieure (IBENS)
Ecole Normale Supérieure, Université PSL, CNRS, INSERM, Paris, France

[b] Institut du Fer à Moulin (IFM)
INSERM, Sorbonne Université, Paris, France

[c] Central Nervous System Diseases Research
Boehringer Ingelheim Pharma GmbH & Co. KG, Biberach an der Riss, Germany

* Correspondence to Dr. Pierre Paoletti: pierre.paoletti@ens.psl.eu

This PDF file includes:

Supplementary Methods

Figures S1 to S6

Table S1

Supplementary Methods

Animals and Ethical Statement

Mice were housed in the IBENS and IFM rodent facilities accredited by the French Ministry of Agriculture. Mice were maintained in laboratory cages under a 12 h light/dark cycle with food and water *ad libitum*. All experiments were performed in compliance with the French and European regulations on care and protection of laboratory animals (EU Directive 2010/63, French Law 2013-118, February 6th, 2013), and were approved by local ethics committees and by the French Ministry of Research and Innovation (authorization numbers #28867 and #44478). For RNA-scope and electrophysiology experiments, C57BL/6J mice purchased from Charles River Laboratories (Saint-Germain-Nuelles, France) were used. Following delivery to the laboratory, mice were allowed to recover at least one week before experimental use. For experiments involving GluN3A-KO mice, WT littermates were also used as controls. Electrophysiology experiments on SST-INs and PV-INs were performed using Sst-IRES-Cre X Ai9 (RCL-tdT); PV-Cre X Ai9 (RCL-tdT) mouse lines, respectively. Both adult male and adult female mice were used.

For behavior experiments, C57BL/6J male and female mice (8 weeks old) were obtained from Charles River Laboratories and were delivered to the animal facility at least one week before surgery. Animals were housed in standard laboratory cages in groups of 3. All behavioral experiments were performed in a blind manner during the light phase. All efforts were made to minimize the number of animals and their discomfort throughout the experiments.

Brain slice preparation

Unless otherwise specified, adult (>P55) mice were deeply anesthetized with isoflurane and decapitated. The brain was quickly removed and placed in ice-cold sucrose-based ACSF, which contained (in mM): 86 NaCl, 2.5 KCl, 0.5 $CaCl_2$, 7 $MgCl_2$, 1.2 NaH_2PO_4, 25 $NaHCO_3$, 25 glucose and 75 sucrose continuously bubbled with carbogen (95% O_2/ 5% CO_2). Acute coronal slices (300 µm thick) containing the dorsal hippocampus (DH) or horizontal slices (300 µm thick) with the ‘magic cut’ containing the ventral hippocampus (VH) (1) were prepared using a 7000 SMZ-2 Vibratome (Campden Instruments Ltd, UK) in ice-cold sucrose-based ACSF. Slices were transferred for 30

minutes in warm (34°C) ACSF which contained (in mM): 125 NaCl, 2.5 KCl, 2 $CaCl_2$, 1 $MgCl_2$, 1.25 NaH_2PO_4, 25 $NaHCO_3$ and 25 glucose, saturated with 95 % O_2 - 5 % CO_2, and then placed in ACSF at room temperature for the rest of the experiment.

Ex vivo electrophysiology

Brain slices were moved to a recording chamber and continuously perfused with ACSF bubbled with 95% O_2/5% CO_2 (3-4 mL/min; 30-34°C). Recorded neurons were visualized with an Olympus BX51WIF microscope (Olympus, France) equipped with a Qimaging RETIGA 2000R camera (Teledyne Photometrics, USA) run by Micro-Manager (Vale Lab, USCF, USA). For whole-cell experiments and loose cell-attached experiments, borosilicate glass pipettes of 3–5 MΩ resistance were filled with an intracellular solution containing (in mM): 130 K-Gluconate; 0.6 EGTA; 2 $MgCl_2$; 0.2 $CaCl_2$; 10 HEPES; 2 Mg-ATP; 0.3 Na_3-GTP, pH 7.3 with KOH (295-300 mOsm). For whole-cell NMDA puffs experiments, the patch pipettes were filled with (in mM): 120 $CsMeSO_3$; 10 HEPES; 4.6 $MgCl_2$; 10 K2-creatine phosphate; 15 BAPTA; 4 Na_2-ATP; 0.4 Na_2-GTP, 0.05 4-AP and 10 TEA-Cl. For both intracellular solutions, values were not corrected for liquid junction potential. Electrophysiological signals were recorded with a Multiclamp 700B amplifier (CV-7B headstage), a Digidata 1440A acquisition board and pClamp 10.3 software (Molecular Devices, USA). Electrophysiological signals were filtered at 2 kHz and sampled at 10 kHz. Data were analyzed off-line (Clampfit 10.7, Molecular Devices). Series resistances were compensated up to 65% maximum. During puff experiments, Glycine (10 mM or 1 mM) or NMDA (1 mM) were puffed locally near to the recorded cell through a borosilicate glass pipette (2-3 MΩ tip resistance) for 1000-5000 milliseconds with a pneumatic drug ejection system PicoPump PV 820 (WPI, Germany). For glycine puff experiments, puff pipettes and bath solution contained antagonists of $GABA_A$ receptors (10 µM Bicuculine), pentameric glycine receptors (20 µM strychnine), AMPA receptors (10 µM NBQX) and NMDARs (50 µM D-AP5) to isolate GluN1/GluN3A receptor mediated currents, as well as TTX (200 nM). The same cocktail of receptor antagonists was added to the bath solution during holding current experiments. For loose cell-attached experiments, TTX was omitted from the bath solution. EU1180-438 (NAM) (2) 30 µM, and DCKA 500 µM were used to inhibit eGlyR mediated currents. The NAM effect was analyzed after 20 minutes of acute

application once the plateau has been reached. For NMDA puff experiments, strychnine and D-AP5 were omitted from the puff and the bath solutions. The I-V curves were obtained by normalizing the values to the current charge obtained at +40 mV. To test corticosterone (CORT) effect on eGlyR-mediated current, CORT (1 µM) was acutely applied during the glycine puff experiments in the presence of CGP (1 µM). CORT effect was analyzed after 20 minutes of acute application. To probe changes in the excitability of CA1 PNs, a stimulation pipette containing HEPES-buffered solution (HBS) was placed in the *stratum radiatum* of CA1 (stimulation of Shaffer collaterals) while action potentials were recorded in loose cell-attached configuration from CA1 PNs. Extracellular synaptic stimulation (Figure 4D) was obtained using an A-M System model 2100 stimulator and 5 voltage pulses were delivered at 50 Hz with a 30 s interval between each sweep. The number of evoked spikes upon stimulation was calculated by averaging the last 10 sweeps before NAM or CORT application, and 10 sweeps after 20 minutes of NAM or CORT application. LTP recordings were performed using two glass pipettes containing HBS and placed in the *stratum radiatum* of CA1 (stimulation of Shaffer collateral). Stimulation was evoked using an A-M System model 2100 stimulator. LTP was induced by high-frequency stimulation (HFS) delivered at a frequency of 100 Hz (1 s) for three times (20 s interval between each sweep, 1 HFS for sub-optimal protocol). Before applying the HFS, baseline values were recorded at a frequency of 0.05 Hz for at least 10 minutes. Responses were digitized at 2 kHz. Data acquisitions were performed using pClamp 10.3 software (Molecular Devices, USA), off-line analysis using Python script (Neo I0 module). Slope potentiation factors were calculated dividing the average slope value between 45 and 55 minutes after tetanic stimulation by the average slope value of 10 minute of baseline. Where specified, NAM (30 µM) was added to the ACSF solution throughout the entire duration of the LTP experiment. For CORT experiments, slices were pre-incubated with CORT (1 µM) for one hour before LTP induction and the entire LTP protocol was done in presence of CORT.

Cell labelling

To approximate the coordinates and confirm the neuronal cell type, recorded neurons were labelled with neurobiotin 488 tracer (Vector Laboratories). Neurobiotin was diluted in the intracellular solution and a whole-cell patch-clamp recording was performed for at least 20

minutes to allow the diffusion of the tracer. At the end of the recording, an outside-out patch was realized, and the slice immediately transferred in PFA 4% and let at 4°C o/n for the fixation. After three washes in PBS 0.1 M, the slices were permeabilized with a solution containing 0.3% Triton and 0.05% NaN_3 in PBS 0.1 M o/n at room temperature. Staining was performed using a streptavidin-Alexa 488 2 µg/mL and DAPI. Slices were mounted in vectashield and images acquired with a Leica SP8 inverted confocal.

Multi-Electrode Array (MEA) recordings

The spike activity of CA1 subregion of the VH was recorded using a 64-channel multielectrode array (MEA) system (MED64, Alpha MED Scientific). Briefly, brain slices containing the VH were placed on a PEI-coated MEA probe (MED-P515A) and immobilized by a small platinum anchor. Recordings started after a 15 minutes recovery period in ACSF solution. Spiking activity was recorded for 10 minutes in drug-free ACSF as baseline and for 30 minutes upon the application of an ASCF solution containing EU1180-438 (NAM) 30 µM. Extracellular field potentials were filtered with a band-pass filter with cutoff frequencies set at 100 Hz and 10 kHz, and data were acquired at a sampling rate of 20 kHz. Recordings were performed at 37°C. Spike detection was performed using MOBIUS software with a detection threshold set at 6 times the standard deviation of the estimated noise for each electrode. Data analysis was performed using a custom R script available at the following GitLab link: https://gitlab.com/icm-institute/dac/biostats/MEASpikeR. Electrodes that detected at least 1 spike per minute were categorized as active electrodes. For each slice, the mean firing rate (MFR), which represents the average firing rate across all active electrodes, was analyzed for 10 minutes in drug-free ACSF as baseline and between 20 and 30 minutes after NAM application.

RNAscope In Situ Hybridization

In Situ Hybridization was performed using the RNAscope® Multiplex Fluorescent Kit v2 (Advanced Cell Diagnostics) with the following probes: *Grin3A*-C1 (551371), Slc17a7C3 (*vvGlut1;* 16 631-C3), and *Gad1*-C2 (#400951-C2). Successive steps were performed as described in the user manual. In brief, brain slices of 15 µm of thickness were obtained using the cryostat. Slides were dried for

60 min at –20°C, washed in PBS for 5 min, then baked for 10 min at 60◦C. The slides were then postfixed by immersion in 4% PFA for 10 min, washed and dehydrated in 50%, 70%, and 100% ethanol, and dried for 5 min at RT. About 3–5 drops of RNAscope hydrogen peroxide was applied to the slides for 10 min. Slides were transferred to a container with RNAscope target retrieval reagent and incubated at >95°C for 5 min, then incubated in 100% alcohol for 3 min. After drying, they were treated with Protease III for 20 min at 40°C and 1X target probe mixes were applied to the brain sections and incubated at 40°C for 2 h in the HybEZ™ oven (Advanced Cell Diagnostics). Sections were then incubated with preamplifier and amplifier probes and developed HRP-C1, C2, and C3 signals with TSA Plus fluorophores (PerkinElmer). After washing, sections were stained with DAPI. Fluorescence confocal images were captured on the AX Nikon Spatial Array Confocal (*NSPARC*) with a 60x objective. For the quantification of excitatory neurons expressing *Grin3A,* selected ROI were positioned manually on the CA1 or CA3 pyramidal layer using the DAPI signal. The colocalization of *Grin3a*-positive cells with *vGlut1* was quantified using Fiji software and the percentage of excitatory cells (*vGlut1* positive) expressing *Grin3a* was then calculated. For interneurons, all *Gad1* positive cells in the image were considered and the percentage of *Grin3a* positive cells among them was calculated.

Western Blotting

Hippocampi of adult mice (P65) were dissected and the dorsal and ventral regions separated and collected. Tissues were lysed in lysis buffer (1 g sucrose; 1.25 ml Tris 0.5 M pH 6.8; 1 mL SDS 10%; 1 mL protease inhibitor cocktail), and centrifuged at 16000 g at 4° for 15 min. Protein content was assessed by Coomassie Protein Assay Kit (Thermo Fisher) and bovine serum albumin-based (BSA) standard curve. For each sample, 15 µg of protein were loaded for WB migration (diluted in reducing Laemmli). Membrane was blocked with TBS-T (20 mM Tris, 150 mM NaCl, 0.15% Tween-20) and 5% milk and incubated over-night at 4° with anti-NR3A rabbit antibody (Millipore 07-356; 1:750) or anti-alpha-tubulin mouse antibody (Millipore, 05-829; 1:1000). HRP-conjugated secondary antibodies anti-rabbit (Jackson 111-035-003) or anti-mouse (Jackson 115-035-003) were used. Protein bands were revealed with SuperSignal™ West Pico PLUS Chemiluminescent Substrate (ThermoFisher 34577).

Viral injections

9-weeks old C57BL6J mice were bilaterally infused in the ventral hippocampus with either AAV9-CMV-EGFP-H1-shRNAGluN3A or AAV9-GFP-H1-scr-shRNA (500 nL at 50 nL/min), manufactured by Vector Biolabs (Marlvern, PA)(3). For eGlyR selective downregulation in SST-INs, AAV9-EF1-mCherry-U6-SICO-GFP-scramble or AAV9-EF1-mCherry-U6-SICO-GFP-shGluN3A were injected in Sst-IRES-Cre mice (P65). The following coordinates relative to Bregma were used: anterior-posterior [AP], -3.2 mm; medial lateral [ML], ±3.2 mm. The dorsoventral [DL], -3.45 mm coordinate was chosen relative to brain surface. Following the injection, the needle was left in place for 5 minutes to allow full diffusion of the viruses. Experiments (*ex vivo* electrophysiology and behavior) were performed 2 to 5 weeks after injection.

Behavioral experiments

A lot of 23 mice (12 females and 11 males) in the Scramble group and 22 mice (11 females and 11 males) in the shGluN3A group have been used. The open field test, the dark-light box and the elevated O-maze were exploited to assess anxiety-like behavior based on the conflict between rodents' tendency to explore a novel environment and the fear of bright and/or open spaces. Animals were also evaluated in the marble burying test, as rodents show burying behavior in the presence of aversive stimuli such as an electric shock and noxious food.

Three weeks after virus injection, animals were tested individually, alternating between males and females. Each mouse was tested on day 1 in the open field test, on day 3 in the dark-light box and marble burying task in the morning, then again in the open field test in the afternoon, and on day 5 in the elevated O-maze in the morning and in the afternoon for the third time in the open field test (see Fig. S6*A*). On a given day, only when all the mice had completed a given test were the animals exposed to another test. Each exploration session lasted 15 minutes and was recorded and post-rated every 5 minutes to assess within-session habituation. Videos were captured at 30 fps with a Basler camera (Basler ace U, acA1300-200 um). Using the position of the animal's center of gravity, the temporal characteristics and spatial distribution of the mice's locomotion were analyzed using ANY-maze software (Stoelting). It allowed the automatic calculation of distance travelled, immobility time, speed, latency of first entry, time spent and

number of entries in different zones of mazes. After each test, the mouse was returned to its home cage and the apparatus was cleaned with water and carefully dried.

The open field test is a brightly lit (~ 600 lux measured in the center) white square arena (50 cm, polyvinyl chloride) divided into three zones (central, intermediate and peripheral). For 3 days, mice were placed on the periphery of the apparatus at the beginning of each session and their activity was automatically recorded.

The dark-light box is a polyvinyl chloride box divided by an open door (8 cm wide) into a white illuminated area (~ 500 lux, 30 x 20 cm) and a dark black area (~ 150 lux, 15 x 20 cm). Mice were placed in the black box and their activity was automatically recorded when the opening was cleared. The number of attempts was scored manually. An attempt was defined as the animal keeping both hind feet in the white area while extending its head and shoulders forward, followed by retraction.

The elevated O-maze test (external diameter 50 cm) consisted of a 5 cm wide annular polyvinyl chloride runway divided into two white open areas and two dark wall-enclosed areas of equal size. Mice were individually released into one of the protected areas and their activity was automatically recorded. The number of unprotected head dips (open arms) was scored manually.

In the marble burying test, mice were individually placed in a transparent acrylic chamber (internal dimensions 25 × 17 × 12 cm, L × l × H) with a smooth transparent plastic lid to prevent the mice from clinging to the chamber lid. The chamber contained 12 spherical glass marbles (diameter: 15 mm) evenly spaced (3 rows of 4 marbles per row) on a 5 cm layer of sawdust (small wood chips of at least 1-2 mm, Lignocel® select, JRS). Mice were left in the chamber with the marbles for 20 minutes and the number of marbles buried in the sawdust was reported every minute. A marble was considered hidden when it was at least two-thirds covered by sawdust. Before testing the next mouse, the sawdust was changed and the marbles were washed with water and dried with paper towels.

Data analysis and statistics

Data were analyzed using the following software: Clampfit 10.7 (Molecular Devices), Excel (Microsoft Office) and Prism 10 (GraphPad Software). Statistical tests were performed with Prism. For *ex-vivo* experiments, two-tailed nonparametric Mann–Whitney test and Kruskal-Wallis followed by Dunn's multiple comparisons test were performed when groups were not large enough to accurately verify normality and equality of variance or when data were not normally distributed. The 1-sample Wilcoxon's signed ranks non-parametric test was used to evaluate the effect of CGP-78608 and DCKA on holding currents. Two-way ANOVA followed by Tukey's or Sidak's multiple comparisons test was also used when appropriate. When datasets followed a normal distribution, paired t-tests and one-way ANOVA tests were used. For behavioral experiments, repeated measures designs were analyzed using a mixed effect restricted maximum likelihood (REML) model, with independent variables (such as period, day, or treatment, depending on the experimental design) as fixed effects and subject as a random effect. The normality of the residuals was confirmed graphically using the diagnostic plots method. The significant main effects were further analyzed by *post hoc* comparison of means using Sidak's multiple comparison test. The Mann-Whitney test was used to analyze between-subjects designs. Statistical significance was set at $p<0.05$ (*$P<0.05$, **$P<0.01$, ***$P<0.001$). Data are expressed as mean ± SEM.

References

1. J. Bischofberger, D. Engel, L. Li, J. R. Geiger, P. Jonas, Patch-clamp recording from mossy fiber terminals in hippocampal slices. *Nat Protoc* **1**, 2075–2081 (2006).
2. Z. Zhu, *et al.*, Negative allosteric modulation of GluN1/GluN3 NMDA receptors. *Neuropharmacology* **176**, 108117 (2020).
3. S. Marco, A. Murillo, I. Pérez-Otaño, RNAi-Based GluN3A Silencing Prevents and Reverses Disease Phenotypes Induced by Mutant huntingtin. *Molecular Therapy* **26**, 1965–1972 (2018).

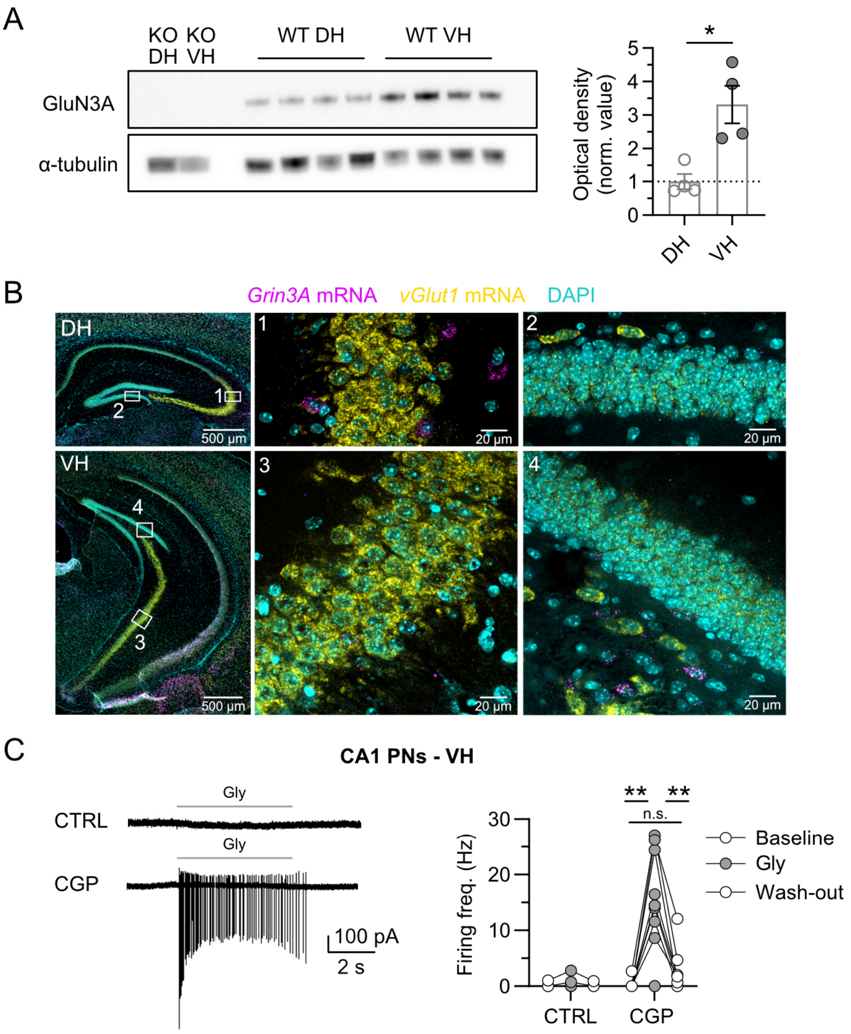

Fig. S1: Differential expression of eGlyRs between the dorsal and ventral hippocampus and cell-type specific expression in the ventral hippocampus. *(A)* Immunoblots of GluN3A protein in the dorsal and ventral regions of the adult (P65) mouse hippocampus. GluN3A shows a much higher expression in the ventral hippocampus (VH) compared to the dorsal hippocampus (DH) (WT DH [n = 4] vs WT VH [n = 4], p = 0.0286; Mann-Whitney test). GluN3A KO tissues were used as negative controls. ***(B)*** Coronal sections of the dorsal hippocampus (DH) and ventral hippocampus (VH) labeled for *Grin3a* mRNA (magenta), *vGlut1* mRNA (yellow) and DAPI (cyan). High-magnification images of the boxed areas in CA3 and DG of DH (1, 2) and VH (3, 4). ***(C)*** Loose cell-attached experiments in PNs of the VH with and without CGP (CTRL [n = 11]: Baseline vs Gly p = 0.4009, Baseline vs Wash-out p = 0.5934, Gly vs Wash-out p = 0.4058; CGP [n = 9]: Baseline vs Gly p = 0.0015, Baseline vs Wash-out p = 0.2028, Gly vs Wash-out 0.0015; two-way ANOVA followed by Tukey's multiple comparisons test). Bars indicate mean ± SEM.

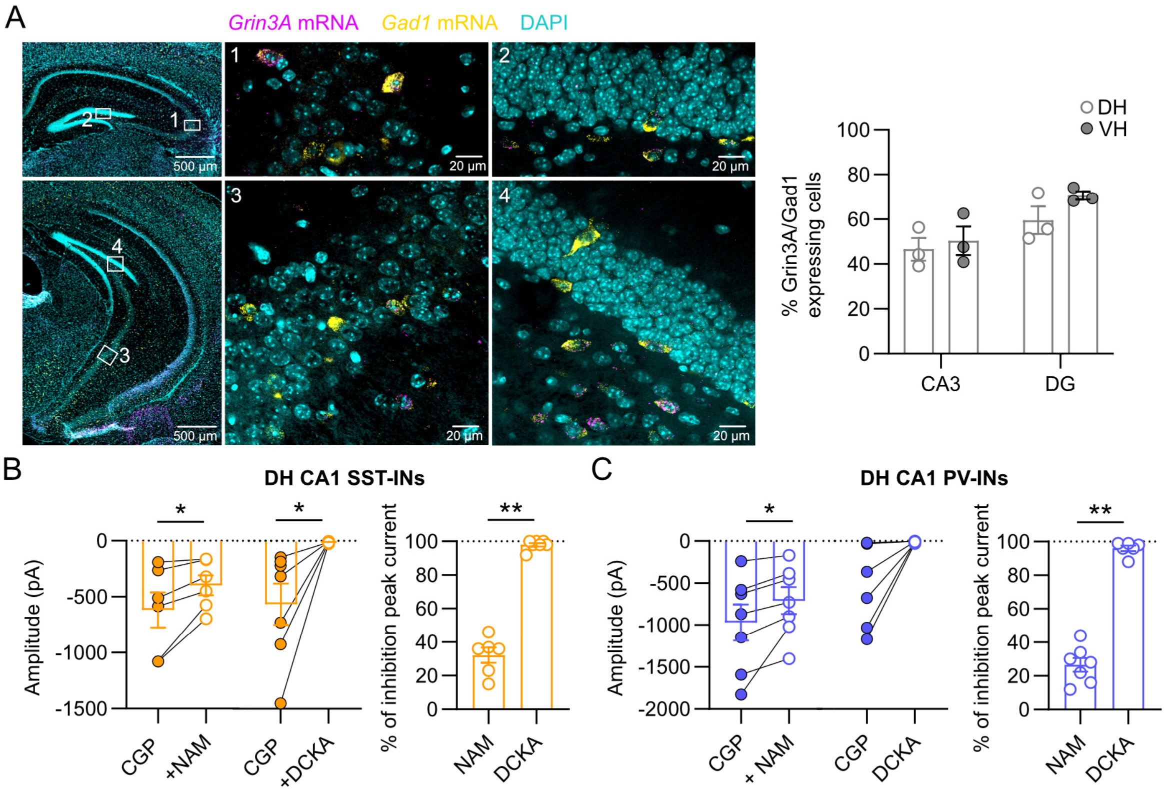

Fig. S2. Widespread expression of eGlyRs in interneurons of the dorsal and ventral hippocampus. *(A)* (left): mosaic images representing the DH and VH labeled for *Grin3a* mRNA (magenta), *Gad1* mRNA (yellow) and DAPI (cyan). (Right): high-magnification images of the boxed areas in CA3 and DG of DH (1, 2) and VH (3, 4) and quantification of the percentage of inhibitory neurons expressing *Gad1*, which express *Grin3a* in CA3 and DG of DH and VH (N = 3 animals and n = 2 fields per condition). ***(B)*** Inhibition of glycine-induced currents (glycine 1 mM, -65 mV) in SST-INs of the DH by NAM (30 µM) and DCKA (500 µM) (CGP vs CGP + NAM [n = 6], p = 0.0313; CGP vs CGP + DCKA [n = 7], p = 0.0156, Wilcoxon test). Right: Percentage of inhibition of the peak current (NAM [n = 6] vs DCKA [n = 7], p = 0.0012, Mann Whitney test). ***(C)*** As *(B)* but for DH CA1 PV-INs (CGP vs CGP + NAM [n = 7], p = 0.0156; CGP vs CGP + DCKA [n = 6], p = 0.0312, Wilcoxon test). Right: Percentage of inhibition of the peak current (NAM [n = 6] vs DCKA [n = 7], p = 0.0012, Mann Whitney test). Bars indicate mean ± SEM.

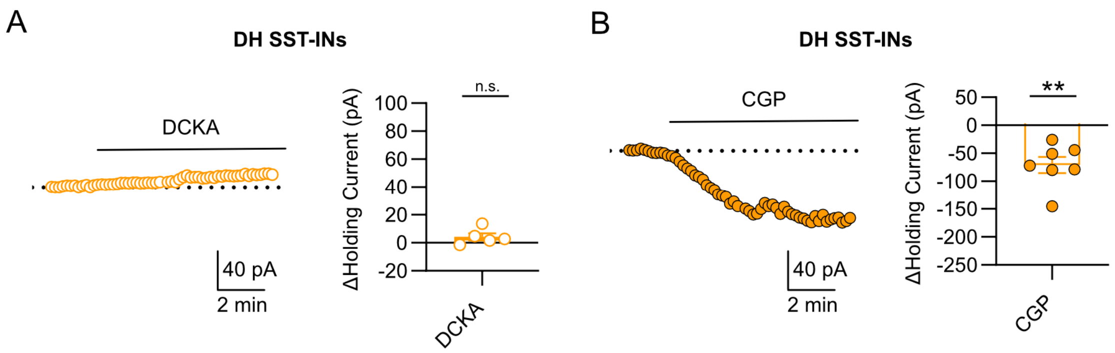

Fig. S3. Tonic activation of eGlyRs in SST-INs of the dorsal hippocampus. ***(A)*** Effect of DCKA application (500 µM) on holding currents in SST-INs of the DH (DCKA [n = 5], p = 0.1708, One-sample Wilcoxon test). ***(B)*** Effect of CGP (1 µM) on inward holding currents in SST-INs of the DH (CGP [n = 7] p = 0.0027, One-sample Wilcoxon test). Bars indicate mean ± SEM.

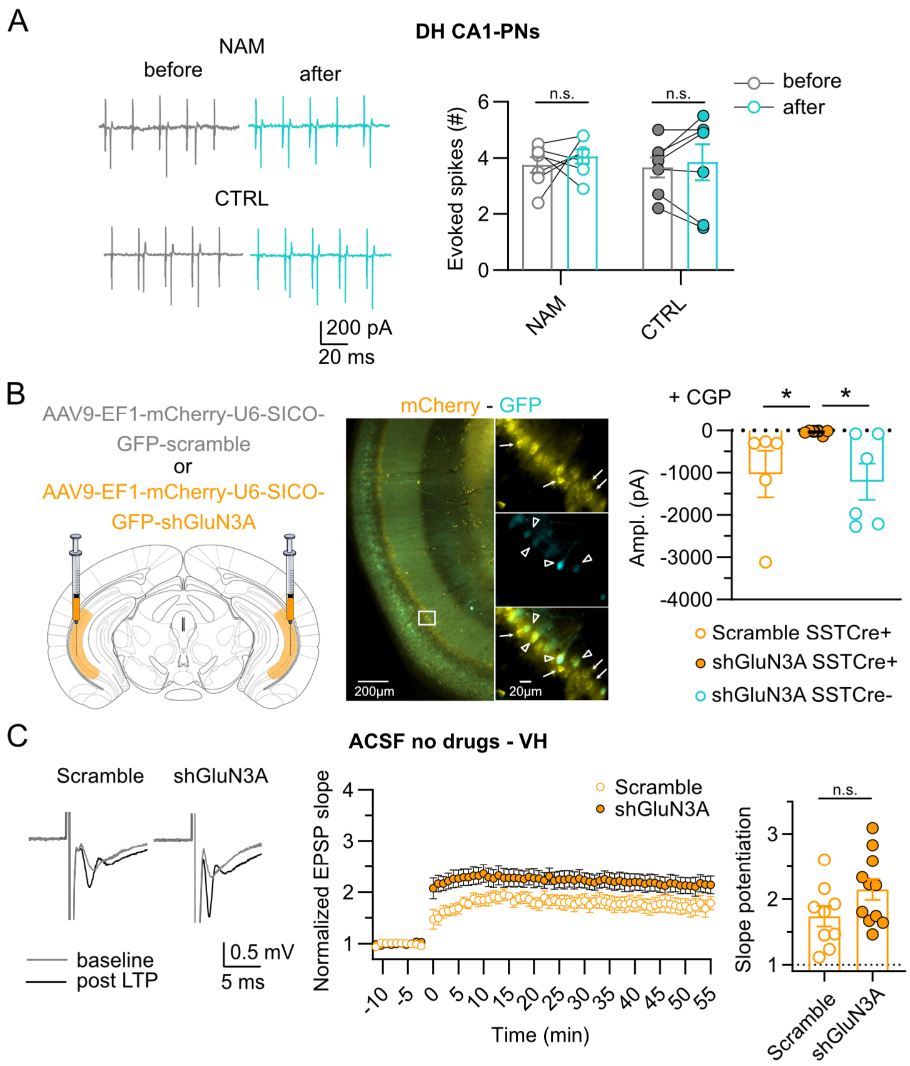

Fig. S4. Inhibition of eGlyRs has no effect on CA1-PN excitability in the dorsal hippocampus and effect of eGlyR downregulation in SST-INs on LTP in the ventral hippocampus. ***(A)*** No effect of NAM (30 µM) application on the firing of CA1 PNs of the dorsal hippocampus (DH) in response to extracellular stimulation (NAM [n = 7]: before vs after, p = 0.7209; CTRL [n = 7] before vs after, p = 0.8801; two-way ANOVA followed by Sidak's multiple comparisons test). ***(B)*** (left): sites in the VH used for bilateral viral injections of either AAV9-EF1-mCherry-U6-SICO-GFP-scramble (scramble) or AAV9-EF1-mCherry-U6-SICO-GFP-shGluN3A (shGluN3A). Infected SSTCre+ cells are mCherry positive and GFP negative (solid arrows), infected SSTCre- cells are mCherry positive and GFP positive (empty arrowheads). (Right): glycine-evoked currents (glycine 1 mM, -65 mV) in CGP (1 µM) are fully absent in shGluN3A SSTCre+ neurons compared to scramble and to shGluN3A SSTCre- 3 weeks post injection (scramble SSTCre+ [n = 5] vs shGluN3A SSTCre+ [n = 6], p = 0.0227; shGluN3A SSTCre+ [n = 6] vs shGluN3A SSTCre- [n = 6], p = 0.0216; scramble SSTCre+ [n = 5] vs shGluN3A SSTCre- [n = 6], p >0.9999; Kruskal-Wallis test followed by Dunn's multiple comparisons test). ***(C)*** LTP in VH of Cre-dependent shGluN3A in SSTCre+ animals vs Scramble (Scramble [n = 9]

vs shGluN3A [n = 11], p = 0.0903; Unpaired t test). In the representative traces the stimulus artifact was cut. Bars indicate mean ± SEM.

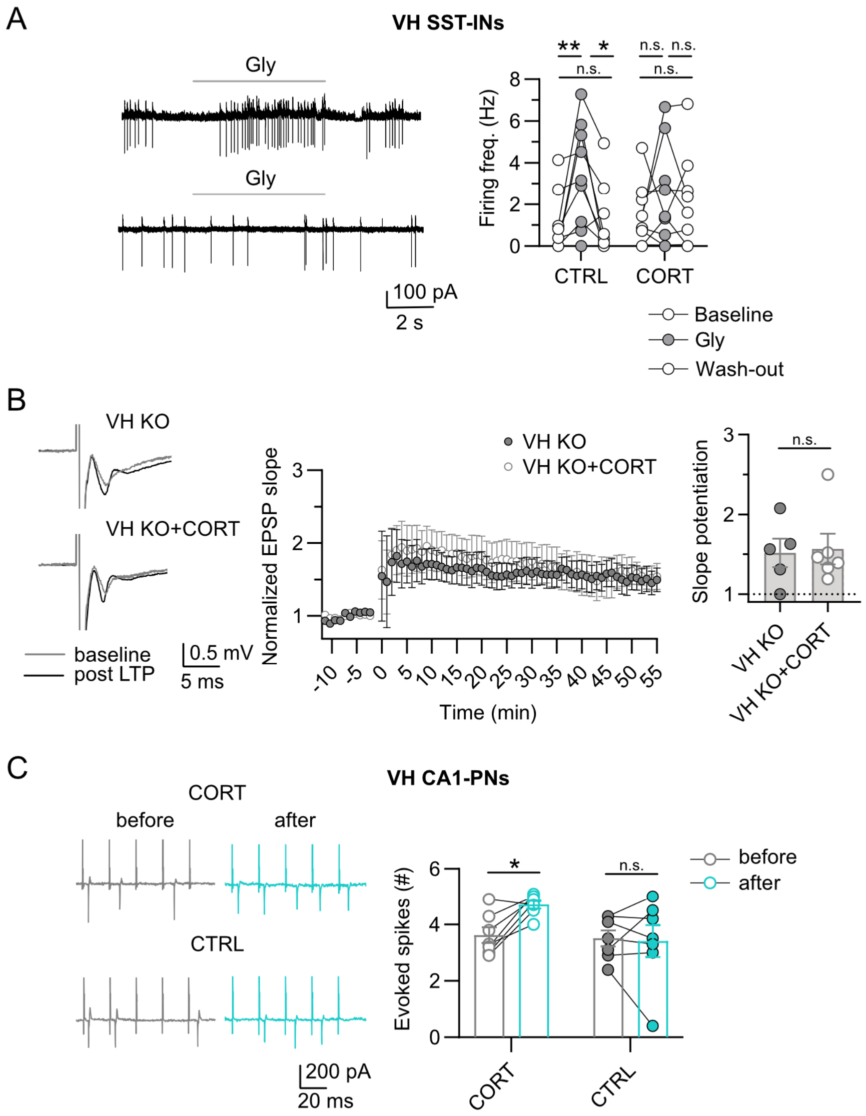

Fig. S5. Corticosterone in the ventral hippocampus prevents the glycine-mediated enhancement of SST-IN firing, does not modulate LTP in GluN3A KO slices, and increases the excitability of CA1-PNs. ***(A)*** Effect of glycine application (1 mM) on the firing of SST-INs of the VH in CTRL and with corticosterone 1 µM (CORT). Loose cell-attached experiments (CTRL [n = 9]: Baseline vs Gly p = 0.0074, Baseline vs Wash-out p = 0.9786, Gly vs Wash-out p = 0.0123; CORT [n = 10]: Baseline vs Gly p = 0.5578, Baseline vs Wash-out p = 0.8324, Gly vs Wash-out 0.8901; two-way ANOVA followed by Tukey's multiple comparisons test). ***(B)*** Corticosterone (CORT 1 µM) effect on VH LTP in GluN3A KO animals after a sub-maximal LTP induction protocol (1s HFS instead of 3 x 1s HFS). VH KO [n = 5] vs VH KO + CORT [n =6], p = 0.9307; Mann-Whitney test. In the representative traces the stimulus artifact was cut. ***(C)*** CORT (1 µM) enhances the firing of CA1 PNs of the VH in response to extracellular synaptic stimulation (CORT [n = 7]: before vs after,

p = 0.0177; CTRL [n = 7] before vs after, p = 0.9511; two-way ANOVA followed by Sidak’s multiple comparisons test). Bars indicate mean ± SEM.

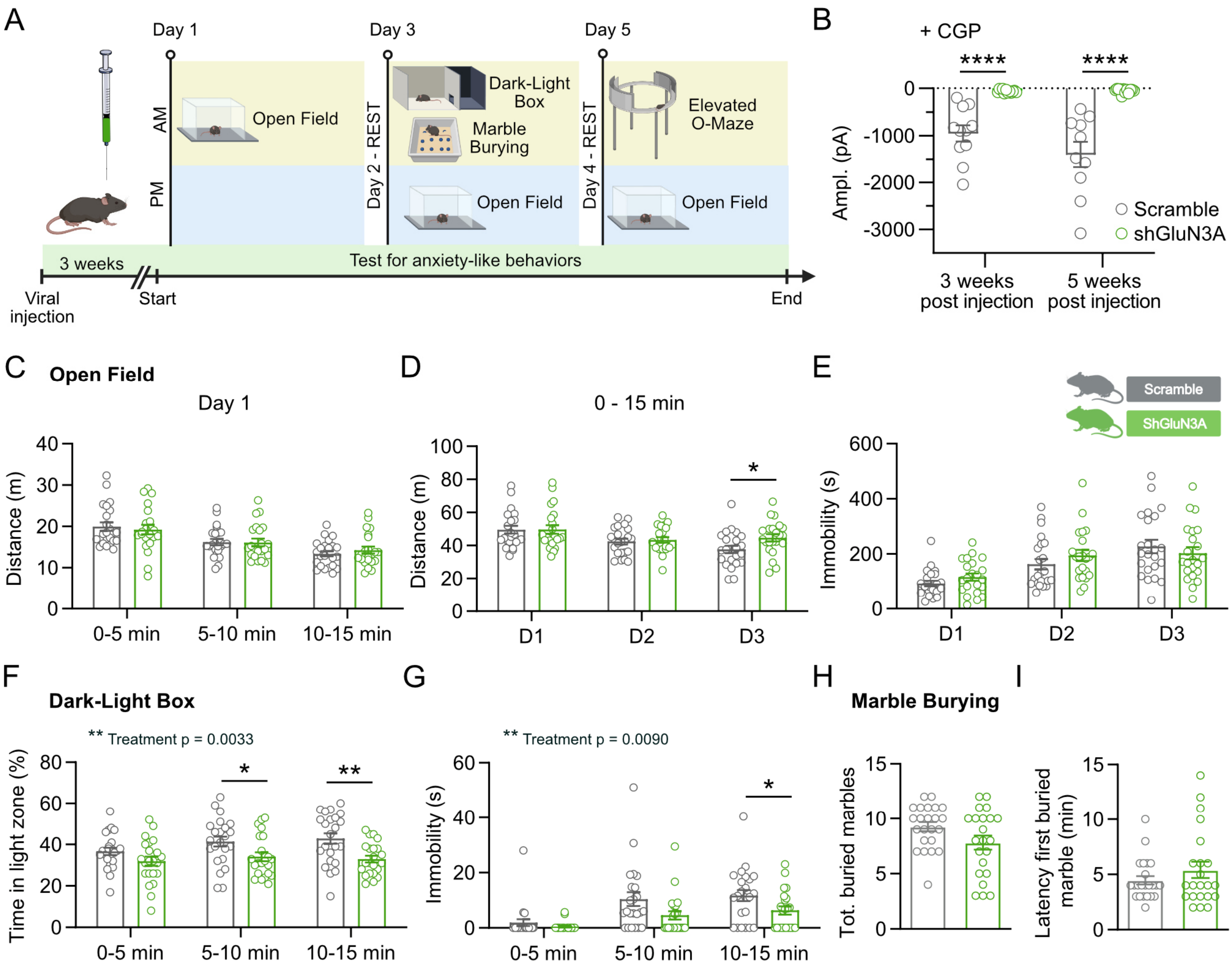

Fig. S6. EGlyRs in the ventral hippocampus participate to anxiety-related behaviors. ***(A)*** Schematic representation of the behavioral experiments' timeline. ***(B)*** Glycine-evoked currents (glycine 1 mM, -65 mV) in CGP (1 µM) are fully absent in shGluN3A GFP-positive cells three-weeks and five-weeks post viral injection compared to scramble (3 weeks post injection: scramble [n = 11] vs shGluN3A [n = 12] $p < 0.0001$, Unpaired t-test; 5 weeks post injection: scramble [n = 10] vs shGluN3A [n = 12] $p < 0.0001$, Mann-Whitney test). ***(C)*** Analysis of the distance travelled on day 1 in the open field test (OF) (Period $F(2, 86) = 4.396$, $p = 16.90$; Treatment $F(1, 43) = 0.00037$, $p = 0.9848$; Period x Treatment $F(2, 86) = 1.342$, $p = 0.2667$). ***(D)*** Total distance travelled in the 15 min OF test from day 1 (D1) to day 3 (D3) (Day $F(2, 86) = 16.90$, $p < 0.0001$; Treatment $F(1, 43) = 1.206$, $p = 0.2783$; Day x Treatment ($F(2, 86) = 3.020$, $p = 0.0540$; D3: shGluN3A vs Scramble $p = 0.0289$). ***(E)*** Immobility in the OF did not change in the two groups (Day $F(2, 86) = 35.40$, $p < 0.0001$; Treatment $F(1, 43) = 0.2328$, $p = 0.2328$; Day x Treatment ($F(2, 86) = 2.630$, $p = 0.0779$). ***(F)*** shGluN3A mice spent less time in the light area compared to controls in the DL test (Period $F(2, 86) = 2.855$, $p = 0.0648$; Treatment $F(1, 43) = 9.703$, $p = 0.0033$; Period x Treatment $F(2, 86)$

= 1.132, p = 0.3272; 5-10 min: shGrin3a vs scramble p = 0.0288; 10-15 min: shGrin3a vs scramble p = 0.0025). ***(G)*** Immobility time in the light compartment (Period F(2, 86) = 13.76, p < 0.0001; Treatment F(1, 43) = 7.480, p = 0.0090; Period x Treatment F(2, 86) = 1.165, p = 0.3168; 10-15 min: shGrin3a vs scramble p = 0.0411). ***(H)*** Scramble and shGluN3A groups showed the same total number of buried marbles in the 20 min test (p = 0.1208, Mann-Whitney test)**.** ***(I)*** Same latency of the first buried marble in shGluN3A vs controls (p = 0.7771, Mann-Whitey test). Points represent mean ± SEM; Scramble N = 23 vs ShGluN3A N = 22 for each test; bars indicate mean ± SEM. Except where otherwise specified, a mixed effect restricted maximum likelihood (REML) model has been used followed by Sidak's multiple comparison test.

Table S1. Values, n and statistical tests

Figure #	Experiment	Mean ± SEM	Animal (N), cell / slice (n)	Statistics
1B	RNA scope<br>% *Grin3a/vGlut1* expressing cells	DH = 14.06 ± 0.6597<br>VH = 90.98 ± 2.424	N = 3 animals, n = 2 fields for each condition	
1D	Glycine puffs CA1 PNs	DH = -13.12 ± 8.750<br>VH = -611.4 ± 120.6<br>VH GluN3A KO = 5.094 ± 3.170	DH [N = 4, n = 9]; VH [N = 3, n = 9]; VH GluN3A KO [N = 3, n = 7]	Kruskal-Wallis followed by Dunn's multiple comparisons test<br>DH vs VH p = 0.0118; VH vs VH GluN3 KO p < 0.0001
1E (left)	NAM and DCKA inhibition of eGlyRs in<br>VH CA1-PNs	CGP = -292.5 ± 81.72<br>+ NAM = -190.5 ± 59.95<br><br>CGP= -237.9 ± 58.65<br>+ DCKA = -3.126 ± 0.3087	CGP vs CGP + NAM [N = 4, n = 7];<br>CGP vs CGP + DCKA [N = 2, n = 5]	Paired t test<br>CGP vs CGP + NAM p = 0.0044;<br>CGP vs CGP + DCKA p = 0.0161
1E (right)	% inhibition peak current	NAM = 39.61 ± 5.501<br>DCKA = 98.20 ± 0.3742	NAM [N = 4, n = 7];<br>DCKA [N = 2, n = 5]	Unpaired t test<br>p < 0.0001
1F	Glycine puffs CA1 PNs, CA3 PNs, GC<br>of VH	CA1 PN = -229.5 ± 31.84<br>CA3 PN = -2.751 ± 1.932<br>GC = -9.088 ± 5.710	CA1 PNs [N = 7, n = 14]; CA3 PN [N = 4, n = 7];<br>GC [N = 3, n = 7]	Ordinary one-way ANOVA followed by Holm-Sidak's multiple comparisons test<br>CA1 PNs vs CA3 PN p < 0.0001;<br>CA1 PNs vs GC p < 0.0001
1H	NMDA puffs	<u>-60 mV</u>:<br>WT VH = -0.1527 ± 0.02911<br>WT DH = -0.1689 ± 0.009263<br>KO VH = -0.1197 ± 0.01533<br><br><u>-80 mV</u>:<br>WT VH = -0.07811 ± 0.01487<br>WT DH = -0.07717 ± 0.01929<br>KO VH = -0.09872 ± 0.02824	WT VH [N = 4, n = 9];<br>WT DH [N = 4, n = 6];<br>KO VH [N = 3, n = 7]	Kruskal-Wallis followed by Dunn's multiple comparisons test<br><u>-60 mV</u>: WT VH vs WT DH p = 0.9662;<br>WT VH vs KO VH p = 0.7450; WT DH vs KO VH, p = 0.1419;<br><u>-80 mV</u>: WT VH vs WT DH, p > 0.9999; WT VH vs KO VH p > 0.9999; WT DH vs KO VH p > 0.9999;

2B	RNA scope<br>% *Grin3a/Gad1* expressing cells	DH = 69.03 ± 3.588<br>VH = 54.55 ± 3.370	N = 3 animals, n = 2 fields for each condition	
2D	Glycine puff SST-INs DH vs VH	DH: Ctrl = -20.636 ± 14.5518<br>CGP = -437.178 ± 89.3097<br>VH: Ctrl = -60.851 ± 29.1734<br>CGP = -534.886 ± 203.9406	VH [N = 5, n = 10]; DH [N = 5, n = 10]	Two-way ANOVA followed by Sidak's multiple comparisons test<br>VH: CTRL vs CGP p = 0.0116; DH: CTRL vs CGP p = 0.0262
2E (left)	NAM and DCKA inhibition of eGlyRs in VH SST-INs	CGP = -992.2 ± 291.8<br>+ NAM = -681.1 ± 208.1<br><br>CGP= -687.0 ± 167.1<br>+ DCKA = -7.925 ± 2.478	CGP vs CGP + NAM [N = 3, n = 7]; CGP vs CGP + DCKA [N = 4, n = 9]	Paired t test<br>CGP vs CGP + NAM p = 0.0241; CGP vs CGP + DCKA p = 0.0034
2E (right)	% inhibition peak current	NAM = 29.55 ± 5.049<br>DCKA = 100.3 ± 1.828	NAM [N = 3, n = 7]; DCKA [N = 4, n = 9]	Mann Whitney test NAM vs DCKA p = 0.0002
2F	Cell attached firing activity of SST-INs in VH	CTRL: Baseline = 0.897 ± 0.432; Gly = 2.178 ± 0.895; Wash-out = 1.282 ± 0.678<br><br>CGP: Baseline = 0.528 ± 0.277; Gly = 17.779 ± 3.158; Wash-out = 3.238 ± 1.303	CTRL [N = 3, n = 16]; CGP [N = 3, n = 11]	Two-way ANOVA followed by Tukey's multiple comparisons test<br>CTRL: Baseline vs Gly p = 0.0471; Baseline vs Wash-out p = 0.3357; Gly vs Wash-out p = 0.0248;<br>CGP: Baseline vs Gly p = 0.0007; Baseline vs Wash-out p = 0.1128; Gly vs Wash-out p = 0.0007
2H	Glycine puff PV -INs DH vs VH	DH: Ctrl = -16.034 ± 5.698<br>CGP = -280.828 ± 79.841<br>VH: Ctrl = -29.602 ± 20.300<br>CGP = -352.437 ± 91.351	DH [N = 4, n = 10]; VH [N = 3, n = 8]	Two-way ANOVA followed by Sidak's multiple comparisons test<br>DH: CTRL vs CGP p = 0.0061; VH: CTRL vs CGP p = 0.0031
2I (left)	NAM and DCKA inhibition of eGlyRs in VH PV-INs	CGP = -455.7 ± 143.9<br>+ NAM = -327.4 ± 110.9<br><br>CGP = -186.0 ± 47.25	CGP vs CGP + NAM [N = 3, n = 7]; CGP vs CGP + DCKA [N = 2, n = 6]	Wilcoxon test<br>CGP vs CGP + NAM p = 0.0156; CGP vs CGP + DCKA p = 0.0312

		+ DCKA = -5.930 ± 2.025		
2I (right)	% inhibition peak current	NAM = 29.86 ± 2.283<br>DCKA = 97.00 ± 0.3651	NAM [N = 3, n = 7]; DCKA [N = 2, n = 6]	Unpaired t test<br>NAM vs DCKA p < 0.0001
2J	Cell attached firing activity of PV-INs in VH	CTRL: Baseline = 0.897 ± 0.432; Gly = 2.178 ± 0.895; Wash-out = 1.282 ± 0.678<br><br>CGP: Baseline = 0.528 ± 0.277; Gly = 17.779 ± 3.158; Wash-out = 3.238 ± 1.303	CTRL [N = 4, n = 16]; CGP [N = 4, n = 11]	Two-way ANOVA followed by Tukey's multiple comparisons test<br>CTRL: Baseline vs Gly p = 0.1305, Baseline vs Wash-out p = 0.2750, Gly vs Wash-out p = 0.1174;<br>CGP: Baseline vs Gly p = 0.0206, Baseline vs Wash-out p = 0.4049, Gly vs Wash-out 0.0166
3B	DCKA effect on holding current in VH CA1-PNs	WT = 26.21 ± 9.275<br>GluN3A KO = 4.038 ± 1.415	WT [N = 4, n = 10]; GluN3A KO [N = 2, n = 6]	Mann-Whitney test<br>p = 0.0420
3C	CGP effect on holding current in VH CA1-PNs	CGP = 1.395 ± 4.075	N = 4, n = 6	One-sample Wilcoxon test<br>p > 0.9999
3D (left)	DCKA effect on holding current in VH SST-INs	DCKA = 70.52 ± 28.85	N = 4, n = 9	One-sample Wilcoxon test p = 0.0078
3D (right)	DCKA effect on holding current in VH SST-INs Str Pyr vs Str Or	Str Pyr = 92.01 ± 28.73<br>Str Or = 67.47 ± 49.78	Str Pyr [N = 4, n = 5] ; Str Or [N = 4, n = 5]	Mann Whitney test<br>p = 0.3095
3E (left)	CGP effect on holding current in VH SST-INs	CGP = -34.29 ± 13.47	N = 6, n = 17	One-sample Wilcoxon test<br>p = 0.0002
3E (right)	CGP effect on holding current in VH SST-INs Str Pyr vs Str Or	Str Pyr = -8.320 ± 2.381<br>Str Or = -85.56 ± 36.98	Str Pyr [N = 4, n = 6] ; Str Or [N = 4, n = 6]	Mann Whitney test<br>p = 0.0173
3F (left)	DCKA effect on holding current in VH PV-INs	DCKA = 16.73 ± 5.460	N = 4, n = 10	One-sample Wilcoxon test<br>p = 0.0039
3F (right)	DCKA effect on holding current in VH PV-INs Str Pyr vs Str Or	Str Pyr = 12.45 ± 3.750<br>Str Or = 23.15 ± 12.87	Str Pyr [N = 4, n = 6] ; Str Or [N = 4, n = 4]	Mann Whitney test<br>p = 0.9143
3G (left)	CGP effect on holding current in VH PV-INs	CGP = -14.14 ± 4.255	N = 5, n = 14	One-sample Wilcoxon test<br>p = 0.0023

3G (right)	CGP effect on holding current in VH PV-INs Str Pyr vs Str Or	Str Pyr = -6.102 ± 2.787<br>Str Or = -22.17 ± 7.012	Str Pyr [N = 5, n = 7] ; Str Or [N = 5, n = 7]	Mann Whitney test<br>p = 0.0262
4B	NAM effect on LTP of the VH and DH	DH = 2.138 ± 0.1718<br>DH + NAM = 2.154 ± 0.1507<br>VH = 1.540 ± 0.1919<br>VH + NAM = 2.121 ± 0.1297	DH [N = 5, n = 9]; DH+NAM [N = 3, n = 7]; VH [N = 7, n = 10]; VH+NAM [N = 5, n = 9]	Kruskal-Wallis test followed by Dunn's multiple comparisons test<br>DH vs DH+NAM p > 0.9999; VH vs VH+NAM p = 0.0440, DH vs VH p = 0.0338
4C	NAM effect on LTP of the VH in Bicuculline	VH = 1.630 ± 0.1373<br>VH + NAM = 1.338 ± 0.08904	VH [N = 4, n = 8]; VH+NAM [N = 4, n = 7]	Unpaired t test<br>p = 0.1077
4D	VH CA1-PNs excitability upon NAM application	NAM: before = 2.667 ± 0.365; after = 4.3 ± 0.352<br><br>CTRL: before = 2.667 ± 0.295; after = 2.050 ± 0.507	NAM [N = 3, n = 6]; CTRL [N = 3, n = 6]	Two-way ANOVA followed by Sidak's multiple comparisons test<br>NAM: before vs after p = 0.0138;<br>CTRL: before vs after p = 0.4071
4F	Mean firing rate of VH CA1 upon NAM application (MEA experiment)	WT: baseline = 0.210 ± 0.127; NAM = 0.360 ± 0.172<br><br>GluN3A KO: baseline = 0.051 ± 0.019; NAM = 0.060 ± 0.013	WT [N = 5, n = 8]; KO [N = 3, n = 6]	Two-way ANOVA followed by Sidak's multiple comparisons test<br>WT: baseline vs NAM, p = 0.0442; GluN3A GluN3A KO: baseline vs NAM, p = 0.9888
5A (left)	CORT effect on eGlyRs-mediated currents in VH CA1-PNs	CGP = -687.0 ± 121.1<br>CGP+CORT = -473.6 ± 80.49	[N = 5, n = 9]	Paired t test<br>CGP vs CGP + CORT p = 0.0058
5A (right)	% inhibition of peak current	CORT = 29.33 ± 3.919	[N = 5, n = 9]	One sample Wilcoxon test<br>p = 0.0039
5B (left)	CORT effect on eGlyRs-mediated	CGP = -1076 ± 306.5<br>CGP+CORT = -749.5 ± 216.3	[N = 3, n = 7]	Paired t test<br>p = 0.0208

	currents in VH SST-INs			
5B (right)	% inhibition of peak current	CORT = 29.14 ± 5.902	[N = 3, n = 7]	One sample Wilcoxon test<br>P = 0.0312
5C	CORT effect on LTP in VH and VH KO	VH = 1.516 ± 0.09659;<br>VH + CORT = 2.309 ± 0.2561;<br>VH KO = 2.166 ± 0.2152;<br>VH KO + CORT = 1.860 ± 0.1573	VH [N = 6, n = 9]; VH+CORT [N = 6, n = 9]; VH KO [N = 5, n = 9]; VH KO + CORT [N = 6, n = 10]	Kruskal-Wallis test followed by Dunn's multiple comparisons test<br>VH vs VH+CORT p = 0.0253; VH vs VH KO p = 0.0392; VH KO vs VH KO + CORT p > 0.9999
6B	Center time in Open Field	Scramble = 6.539 ± 0.9854; ShGluN3A = 4.836 ± 1.200	scramble N = 23<br>shGluN3A N = 22	Mann-Whitney test<br>p = 0.0374
6C	Open Field distance D1-D3 (0-5 min)	D1: Scramble = 19.956 ± 1.034<br>shGluN3A = 19.239 ± 1.152<br>D2: Scramble = 17.295 ± 0.872<br>shGluN3A = 18.262 ± 0.795<br>D3: Scramble = 14.974 ± 1.112<br>shGluN3A = 19.939 ± 1.181	scramble N = 23<br>shGluN3A N = 22	Mixed effect restricted maximum likelihood (REML) model followed by Sidak's multiple comparison test<br>Day F(2, 86) = 5.063, p = 0.0092; Treatment F(1, 43) = 2.106; Day x Treatment F(2, 86) = 8.088, p = 0.0006;<br>D3: shGluN3A vs Scramble p = 0.0038
6D	Time in the light zone (%)	Scramble = 40.30 ± 1.751; ShGluN3A = 33.09 ± 1.526	scramble N = 23<br>shGluN3A N = 22	Mann-Whitney test<br>p = 0.0030
6E	Number of exits in the DL test	0-5 min : Scramble = 18.913 ± 1.166<br>shGluN3A = 15.409 ± 1.145<br>5-10 min : Scramble = 12.391 ± 0.875<br>shGluN3A = 11.591 ± 0.695<br>10-15 min : Scramble = 11.174 ± 0.670<br>shGluN3A = 9.727 ± 0.614	scramble N = 23<br>shGluN3A N = 22	Mixed effect restricted maximum likelihood (REML) model followed by Sidak's multiple comparison test<br>Period F(2, 86) = 48.13, p < 0.0001; Treatment F(1, 43) = 4.061, p = 0.0502; Period x Treatment F(2, 86) = 1.941, p = 0.1498;<br>0-5 min: shGrin3a vs scramble p = 0.0377
6F	Number of attempts in the DL test	0-5 min : Scramble = 18.913 ± 1.166	Scramble N = 23<br>ShGluN3A N = 22	Mixed effect restricted maximum likelihood (REML) model followed

		shGluN3A = 15.409 ± 1.145<br><u>5-10 min</u> : Scramble = 12.391 ± 0.875<br>shGluN3A = 11.591 ± 0.695<br><u>10-15 min</u> : Scramble = 11.174 ± 0.670<br>shGluN3A = 9.727 ± 0.614		by Sidak's multiple comparison test<br>Period F(2, 86) = 21.62, p < 0.0001; Treatment F(1, 43) = 9.426, p = 0.0037; Period x Treatment F(2, 86) = 0.005, p = 0.9951;<br><u>10-15 min</u>: shGrin3a vs scramble p = 0.0042
6G	Time in open arms (%)	<u>0-5 min</u> : Scramble = 19.087 ± 1.955<br>shGluN3A = 19.636 ± 1.915<br><u>5-10 min</u> : Scramble = 21.826 ± 1.475<br>shGluN3A = 17.591 ± 1.680<br><u>10-15 min</u> : Scramble = 19.522 ± 1.397<br>shGluN3A = 12.682 ± 1.745	Scramble N = 23<br>ShGluN3A N = 22	Mixed effect restricted maximum likelihood (REML) model followed by Sidak's multiple comparison test<br>Period F(2, 86) = 4.396, p = 0.0154; Treatment F(1, 43) = 3.611, p = 0.0641; Period x Treatment F(2, 86) = 3.899, p = 0.0239;<br><u>10-15 min</u>: shGrin3a vs scramble p = 0.0039
6H	Number of Unprotected head dipping	<u>0-5 min</u> : Scramble = 4.565 ± 0.694<br>shGluN3A = 4.727 ± 0.596<br><u>5-10 min</u> : Scramble = 5.043 ± 0.543<br>shGluN3A = 3.182 ± 0.477<br><u>10-15 min</u> : Scramble = 4.087 ± 0.453<br>shGluN3A = 2.000 ± 0.451	Scramble N = 23<br>ShGluN3A N = 22	Mixed effect restricted maximum likelihood (REML) model followed by Sidak's multiple comparison test<br>Period F(2, 86) = 6.256, p = 0.0033; Treatment F(1, 43) = 5.156, p = 0.0282; Period x Treatment F(2, 86) = 3.602, p = 0.0315;<br><u>5-10 min</u>: shGrin3a vs scramble p = 0.0136<br><u>10-15 min</u>: shGrin3a vs scramble p = 0.0022
6I	Buried marbles during time		Scramble N = 23<br>ShGluN3A N = 22	Mixed effect restricted maximum likelihood (REML) model followed by Sidak's multiple comparison test<br>Time F(19, 817) = 192.3, p < 0.0001; Treatment F(1, 43) = 5.087, p = 0.0292; Time x Treatment F(19, 817) = 3.589, p < 0.0001;

S1A	Immunoblots of GluN3A in the dorsal and ventral hippocampus	WT DH = 1.0000 ± 0.2245;<br>WT VH = 3.318 ± 0.5590	WT DH [N = 4];<br>WT VH [N = 4]	Mann-Whitney test<br>p = 0.0286;
S1C	Cell attached firing activity of CA1 PNs in VH	CTRL: Baseline = 0.092 ± 0.092; Gly = 0.323 ± 0.258; Wash-out = 0.085 ± 0.085<br><br>CGP: Baseline = 0.302 ± 0.302; Gly = 15.890 ± 2.969; Wash-out = 2.658 ± 1.270	CTRL [N = 2, n = 11];<br>CGP [N = 2, n = 9]	Two-way ANOVA followed by Tukey's multiple comparisons test<br>CTRL: Baseline vs Gly p = 0.4009, Baseline vs Wash-out p = 0.5934, Gly vs Wash-out p = 0.4058;<br>CGP: Baseline vs Gly p = 0.0015, Baseline vs Wash-out p = 0.2028, Gly vs Wash-out 0.0015
S2A	RNA scope<br>% *Grin3a/Gad1* expressing cells	CA3: DH = 46.55 ± 5.090; VH = 50.39 ± 6.410<br>DG: DH = 59.58 ± 6.223; VH = 70.59 ± 1.784	N = 3 animals, n = 2 fields<br>for each condition	
S2B (left)	NAM and DCKA inhibition of eGlyRs in<br>DH SST-INs	CGP = -616.7 ± 156.8<br>+ NAM = -395.2 ± 88.72<br><br>CGP= -566.1 ± 185.3<br>+ DCKA = -4.071 ± 5.952	CGP vs CGP + NAM [N = 4, n = 6]; CGP vs CGP + DCKA [N = 2, n = 7]	Wilcoxon test<br>CGP vs CGP + NAM p = 0.0312; CGP vs CGP + DCKA p = 0.0156
S2B (right)	% inhibition peak current	NAM = 32.17 ± 4.556<br>DCKA = 98.00 ± 1.069	NAM [N = 4, n = 6];<br>DCKA [N = 2, n = 7]	Mann Whitney test<br>p = 0.0012
S2C (left)	NAM and DCKA inhibition of eGlyRs in<br>DH PV-INs	CGP = -983.1 ± 216.1<br>+ NAM = -722.6 ± 160.3<br><br>CGP= -549.7 ± 201.2<br>+ DCKA = -12.75 ± 5.261	CGP vs CGP + NAM [N = 3, n = 7]; CGP vs CGP + DCKA [N = 2, n = 6]	Wilcoxon test<br>CGP vs CGP + NAM p = 0.0156; CGP vs CGP + DCKA p = 0.0312
S2C (right)	% inhibition peak current	NAM = 26.57 ± 4.134<br>DCKA = 96.00 ± 1.693	NAM [N = 3, n = 7];<br>DCKA [N = 2, n = 6]	Mann Whitney test<br>p = 0.0012
S3A	DCKA effect on holding current in DH SST-INs	DCKA = 4.253 ± 2.551	N = 2, n = 5	One-sample Wilcoxon test<br>p = 0.1708

S3B	CGP effect on holding current in DH SST-INs	CGP = -70.95 ± 14.50	N = 3, n = 7	One-sample Wilcoxon test p = 0.0027
S4A	DH CA1-PNs excitability upon NAM application	NAM: before = 3.757 ± 0.279; after = 4.057 ± 0.254<br><br>CTRL: before = 3.671 ± 0.358; after = 3.857 ± 0.604	NAM [N = 3, n = 7]; CTRL [N = 3, n = 7]	Two-way ANOVA followed by Sidak's multiple comparisons test<br>NAM: before vs after p = 0.7209<br>CTRL: before vs after p = 0.8801
S4B	eGlyRs-mediated current upon viral injection	Scramble SSTCre+ = -1030 ± 549.3<br>shGluN3A SSTCre+ = -37.10 ± 20.16<br>shGluN3A SSTCre- = -1212 ± 430.2	Scramble SSTCre+ [N = 2, n = 5]; shGluN3A SSTCre+ [N = 2, n = 6]; shGluN3A SSTCre- [N, n = 6]	Kruskal-Wallis test followed by Dunn's multiple comparisons test<br>Scramble SSTCre+ vs shGluN3A SSTCre+ p = 0.0227; shGluN3A SSTCre+ vs shGluN3A SSTCre- p = 0.0216; scramble SSTCre+ vs shGluN3A SSTCre- p >0.9999
S4C	LTP in VH of flox-shGluN3A in SSTCre+ animals vs Scramble	Scramble = 1.737 ± 0.1571;<br>shGluN3A = 2.144 ± 0.1606	Scramble [N = 4, n = 9]; shGluN3A [N = 4, n = 11]	Unpaired t test<br>p = 0.0903
S5A	Corticosterone effect on cell attached firing activity of SST-INs in VH	CTRL: Baseline = 1.005 ± 0.490; Gly = 3.438 ± 0.833; Wash-out = 1.154 ± 0.566<br><br>CORT: Baseline = 1.410 ± 0.467; Gly = 2.151 ± 0.756; Wash-out = 1.823 ± 0.702	CTRL [N = 3, n = 9]; CORT [N = 3, n = 10]	Two-way ANOVA followed by Tukey's multiple comparisons test<br>CTRL: Baseline vs Gly p = 0.0074; Baseline vs Wash-out p = 0.9786; Gly vs Wash-out p = 0.0123;<br>CORT: Baseline vs Gly p = 0.5578; Baseline vs Wash-out p = 0.8324; Gly vs Wash-out 0.8901
S5B	Corticosterone effect on VH LTP in GluN3A KO (1 HFS)	VH KO = 1.516 ± 0.1789<br>VH KO + CORT = 1.566 ± 0.1923	VH KO [N= 2, n = 5]<br>VH KO + CORT [N = 2, n =6]	Mann-Whitney test<br>p = 0.9307

S5C	VH CA1-PNs excitability upon CORT application	CORT: before = 3.629 ± 0.281; after = 4.714 ± 0.140<br><br>CTRL: before = 3.514 ± 0.283; after = 3.414 ± 0.569	CORT [N = 4, n = 7]; CTRL [N = 3, n = 7]	Two-way ANOVA followed by Sidak's multiple comparisons test<br>CORT: before vs after p = 0.0177;<br>CTRL: before vs after p = 0.9511
S6B	eGlyRs-mediated current upon viral injection	3 weeks post injection:<br>scramble = -953.7 ± 170.6<br>shGluN3A = -58.10 ± 9.756<br>5 weeks post injection:<br>scramble = -1402 ± 272.2<br>shGluN3A = -49.95 ± 14.01	3 weeks post injection: scramble [N = 2, n = 11]<br>shGluN3A [N = 2, n = 12]<br>5 weeks post injection: scramble [N = 2, n = 10]<br>shGluN3A [N = 2, n = 12]	Unpaired t-test<br>3 weeks post injection: p < 0.0001<br><br>Mann-Whitney test<br>5 weeks post injection: p < 0.0001
S6C	Distance in open field at day 1	0-5 min : Scramble = 19.956 ± 1.034<br>shGluN3A = 19.239 ± 1.152<br>5-10 min : Scramble = 16.261 ± 0.764<br>shGluN3A = 16.127 ± 0.912<br>10-15 min : Scramble = 13.404 ± 0.662<br>shGluN3A = 14.275 ± 0.862	Scramble N = 23<br>ShGluN3A N = 22	Mixed effect restricted maximum likelihood (REML) model followed by Sidak's multiple comparison test<br>Period F(2, 86) = 4.396, p = 16.90; Treatment F(1, 43) = 0.00037, p = 0.9848; Period x Treatment F(2, 86) = 1.342, p = 0.2667
S6D	Open Field distance D1-D3	D1: Scramble = 49.576 ± 2.309<br>shGluN3A = 49.642 ± 2.598<br>D2: Scramble = 42.448 ± 1.731<br>shGluN3A = 43.464 ± 1.665<br>D3: Scramble = 37.673 ± 2.192<br>shGluN3A = 44.672 ± 2.187	scramble N = 23<br>shGluN3A N = 22	Mixed effect restricted maximum likelihood (REML) model followed by Sidak's multiple comparison test<br>Day F(2, 86) = 16.90, p < 0.0001; Treatment F(1, 43) = 1.206, p = 0.2783; Day x Treatment F(2, 86) = 3.020, p = 0.0540;<br>D3: shGluN3A vs Scramble p = 0.0289

S6E	Open Field immobility D1-D3	D1: Scramble = 92.809 ± 10.109<br>shGluN3A = 115.895 ± 13.116<br>D2: Scramble = 162.274 ± 18.897<br>shGluN3A = 194.927 ± 19.849<br>D3: Scramble = 227.461 ± 24.242<br>shGluN3A = 202.700 ± 21.870	scramble N = 23<br>shGluN3A N = 22	Mixed effect restricted maximum likelihood (REML) model followed by Sidak's multiple comparison test<br>Day F(2, 86) = 35.40, p < 0.0001; Treatment F(1, 43) = 0.2328, p = 0.6319; Day x Treatment F(2, 86) = 2.630, p = 0.0779
S6F	Time in the light zone (%) in DL	0-5 min : Scramble = 36.696 ± 1.776<br>shGluN3A = 31.909 ± 2.279<br>5-10 min : Scramble = 41.391 ± 2.411<br>shGluN3A = 34.091 ± 2.143<br>10-15 min : Scramble = 42.870 ± 2.562<br>shGluN3A = 32.955 ± 1.664	Scramble N = 23<br>ShGluN3A N = 22	Mixed effect restricted maximum likelihood (REML) model followed by Sidak's multiple comparison test<br>Period F(2, 86) = 2.855, p = 0.0648; Treatment F(1, 43) = 9.703, p = 0.0033; Period x Treatment F(2, 86) = 1.132, p = 0.3272;<br>5-10 min: shGrin3a vs scramble p = 0.0288<br>10-15 min: shGrin3a vs scramble p = 0.0025
S6G	Immobility time (%) in DL	0-5 min : Scramble = 1.939 ± 1.253<br>shGluN3A = 0.486 ± 0.336<br>5-10 min : Scramble = 10.396 ± 2.497<br>shGluN3A = 4.564 ± 1.532<br>10-15 min : Scramble = 11.722 ± 2.049<br>shGluN3A = 6.341 ± 1.517	Scramble N = 23<br>ShGluN3A N = 22	Mixed effect restricted maximum likelihood (REML) model followed by Sidak's multiple comparison test<br>Period F(2, 86) = 13.76, p < 0.0001; Treatment F(1, 43) = 7.480, p = 0.0090; Period x Treatment F(2, 86) = 1.165, p = 0.3168;<br>10-15 min: shGrin3a vs scramble p = 0.0411
S6H	Tot. buried marbles	Scramble = 9.261 ± 0.4085<br>ShGluN3A = 7.818 ± 0.6333	Scramble N = 23<br>ShGluN3A N = 22	Mann-Whitney test<br>p = 0.1208
S6I	Latency first buried marble	Scramble = 4.478 ± 0.3817<br>ShGluN3A = 5.409 ± 0.7375	Scramble N = 23<br>ShGluN3A N = 22	Mann-Whitey test<br>p = 0.7771
